# Supplementary material for: Thiamine dose response in human milk with supplementation among lactating women in Cambodia: study protocol for a double-blind, four-parallel arm randomised controlled trial
Source: BMJ Open. 2019 Jul 9;9(7):e029255. doi: 10.1136/bmjopen-2019-029255 (PMC6624064; doi:10.1136/bmjopen-2019-029255)
Supplement: Author's manuscript [file bmjopen-2019-029255.draft_revisions.pdf]

BMJ Open is committed to open peer review. As part of this commitment we make the peer review history of every article we publish publicly available.

When an article is published we post the peer reviewers' comments and the authors' responses online. We also post the versions of the paper that were used during peer review. These are the versions that the peer review comments apply to.

The versions of the paper that follow are the versions that were submitted during the peer review process. They are not the versions of record or the final published versions. They should not be cited or distributed as the published version of this manuscript.

BMJ Open is an open access journal and the full, final, typeset and author-corrected version of record of the manuscript is available on our site with no access controls, subscription charges or pay-per-view fees (http://bmjopen.bmj.com).

If you have any questions on BMJ Open's open peer review process please email info.bmjopen@bmj.com

BMJ Open

Thiamine dose response in human milk with supplementation among lactating women in Cambodia: study protocol for a double-blind, four-parallel arm randomized controlled trial

Journal:	*BMJ Open*
Manuscript ID	bmjopen-2019-029255
Article Type:	Protocol
Date Submitted by the Author:	18-Jan-2019
Complete List of Authors:	Whitfield, Kyly; Mount Saint Vincent University<br>Kroeun, Hou; Helen Keller International Cambodia<br>Green, Tim; South Australian Health & Medical Research Institute<br>Wieringa, Frank; Institut de Recherche pour le Développement, IRD/UM/SupAgro, UMR-204 Nutripass<br>Borath, Mam; Cambodia Ministry of Planning, National Sub-Committee for Food Fortification<br>Sophonneary, Prak; Cambodia Ministry of Health, National Nutrition Programme, Maternal and Child Health Centre<br>Measelle, Jeff; University of Oregon, Department of Psychology<br>Baldwin, Dare; University of Oregon, Department of Psychology<br>Yelland, Lisa; South Australian Health & Medical Research Institute; University of Adelaide, School of Public Health<br>Leemaqz, Shalem; South Australian Health & Medical Research Institute<br>Chan, Kathleen; Mount Saint Vincent University<br>Gallant, Jelisa; Mount Saint Vincent University
Keywords:	thiamine (vitamin B1), dose response, human milk, fortification, salt, infant cognitive development

SCHOLARONE™
Manuscripts

Thiamine dose response in human milk with supplementation among lactating women in Cambodia: study protocol for a double-blind, four-parallel arm randomized controlled trial

Kyly C WHITFIELD[1], HOU Kroeun[2], Tim J GREEN[3], Frank WIERINGA[4], MAM Borath[5], SOPHONNEARY Prak[6], Jeff R MEASELLE[7], Dare BALDWIN[7], Lisa N YELLAND[3,8], Shalem LEEMAQZ[3], Kathleen CHAN[1], Jelisa GALLANT[1]

Corresponding Author: Kyly C. Whitfield, Department of Applied Human Nutrition, Mount Saint Vincent University, 166 Bedford Hwy, Halifax, NS B3M 2J6, Canada. kyly.whitfield@msvu.ca, (902) 457-5978.

Author affiliations

1. Department of Applied Human Nutrition, Mount Saint Vincent University, Halifax, Canada
2. Helen Keller International Cambodia, Phnom Penh, Cambodia
3. South Australian Health and Medical Research Institute, Adelaide, South Australia, Australia
4. UMR-204, Institut de recherche pour le développement, UM/IRD/SupAgro, Montpellier, France
5. National Sub-Committee for Food Fortification, Cambodia Ministry of Planning, Phnom Penh, Cambodia
6. National Nutrition Programme, Maternal and Child Health Centre, Cambodia Ministry of Health, Phnom Penh, Cambodia
7. Department of Psychology, University of Oregon, Eugene, Oregon, USA
8. School of Public Health, The University of Adelaide, Adelaide, South Australia, Australia

Word Count: 4,587

Key Words: thiamine (vitamin B1), dose response, human milk, fortification, salt, infant cognitive development

For peer review only

Abstract

Introduction: Thiamine (vitamin B1) deficiency remains a concern in Cambodia where women with low thiamine intake produce thiamine-poor milk, putting their breastfed infants at risk of impaired cognitive development and potentially fatal infantile beriberi. Thiamine fortification of salt is a potentially low-cost, passive means of combating thiamine deficiency, however both the dose of thiamine required to optimize milk thiamine concentrations, as well as usual salt intake of lactating women, are unknown.

Methods and analysis: In this community-based randomized controlled trial, 320 lactating women from Kampong Thom, Cambodia will be randomized to one of four groups to consume one capsule daily containing 0, 1.2, 2.4, or 10 mg thiamine as thiamine hydrochloride, between 2 and 24 weeks postnatal. The primary objective is to estimate the dose where additional maternal intake of thiamine no longer meaningfully increases infant thiamine diphosphate concentrations (ThDP) 24 weeks postnatally. We will collect sociodemographic, nutrition, and health information, a battery of cognitive assessments, maternal and infant venous blood samples (biomarkers: ThDP and transketolase activity), and human milk samples (biomarker: milk thiamine concentrations). All participants and their families will consume study-provided salt *ad libitum* throughout the trial, and we will measure salt disappearance each fortnight. Repeat weighed salt intakes and urinary sodium concentrations will be measured among a sub-set of 100 participants. Parameters of E_{max} dose-response curves will be estimated using non-linear least squares models with both 'intention to treat' and a secondary 'per-protocol' (capsule compliance ≥80%) analyses.

Ethics and dissemination: Ethical approval was obtained in Cambodia (National Ethics Committee for Health Research 112/250NECHR), Canada (Mount Saint Vincent University Research Ethics Committee 2017-141), and the United States (University of Oregon Institutional Review Board 07052018.008). Results will be shared with participants' communities, as well as relevant government and scientific stakeholders via presentations, academic manuscripts, and consultations. ClinicalTrials.gov Identifier: NCT03616288.

Strengths and limitations of this study

- Human milk is thiamine-responsive, however this is the first study to assess the dose of thiamine required to optimize human milk thiamine concentrations, vital information required to inform fortification programs (minimize costs and unnecessary overages).
- Previous work has identified delayed cognitive development among children with low thiamine intake in early life, however this will be the first study to explore cognitive development among infants with controlled thiamine exposures in the first 6 months postnatal.
- We will collect mother's blood, human milk, and infant's blood at 24 weeks postnatal, enabling a robust evaluation of associations between maternal supplementation and these biomarkers.
- The study intervention begins at 2 weeks postnatal, so while randomization should prevent pre-treatment differences between treatment groups, we will not have biochemical thiamine status of the mother during pregnancy, nor the infant at birth.

- Household salt disappearance measures may over- or under-estimate actual usual salt intake as it will not account for wastage, nor salt from food consumed outside the home (e.g. with relatives, purchased at market), respectively.

For peer review only

Background

Thiamine (vitamin B_1) is an essential water-soluble micronutrient required for energy metabolism and nerve impulse conduction [1,2]. Thiamine deficiency was recently called the forgotten disease of Asia [3], as it remains an under-studied public health issue despite evidence of suboptimal thiamine intake or status in the region [4], including Cambodia [5–11]. Thiamine deficiency is of particular concern among lactating women because mothers with poor dietary thiamine intake and/or status produce milk low in thiamine, putting their exclusively breastfed infants at a high risk of developing infantile beriberi [12]. Infantile beriberi presents during the exclusive breastfeeding period and without treatment can result in death within hours of clinical presentation [4,13]. In addition, a growing body of evidence from an unfortunate 'natural experiment' in which thiamine was erroneously omitted from infant formula [14], suggests that thiamine deficiency not severe enough to cause clinical beriberi symptoms can negatively impact cognitive development and functioning [15–17]. To date the precise pathways and mechanisms remain poorly understood, and important questions remain about the timing and levels of deficiency, as well as the ability of prophylactic or therapeutic interventions to prevent or remediate the effects of suboptimal thiamine status on cognitive outcomes in humans [18].

Thiamine is found in pork, whole grains, and legumes [1], and must be consumed routinely due to the relatively short half-life and lack of body storage [4]. Lactating women have an estimated average requirement (EAR) and recommended dietary allowance (RDA) for thiamine of 1.2 and 1.4 mg/day, respectively [19]. For infants aged 0-6 months, an adequate intake (AI) of 0.2 mg/day was derived using the human milk thiamine concentrations from healthy, well-nourished mothers, assuming a daily milk intake of 780 mL [19].

For peer review only

The dietary staple of Cambodia is B-vitamin poor, white, polished rice, which accounts for upwards of 60% of daily energy intake [20]. Given the challenges associated with supplementation and changing dietary patterns (recently reviewed in [4]), fortification, a sustainable, cost-effective, and passive intervention [21–24], is a potentially suitable solution for improving maternal thiamine intake [23]. Thiamine has few technical constraints as a fortificant [21], and there is no tolerable upper intake level (UL) for thiamine because there have been no reports of adverse effects of excess thiamine intake [19,25,26]. We recently demonstrated that maternal consumption of thiamine-fortified fish sauce significantly increased maternal, human milk, and infant thiamine status [27]. However, centrally produced fish sauce may not reach the poorest communities who make their own fish sauce, and consumption of this condiment is not universal in all regions where we find thiamine deficiency [28,29]. Conversely, salt is a common condiment in most regions of the world, and has proven to be a successful global fortification vehicle for iodine [30,31].

The overall aim of this study is to obtain the information necessary to formulate a thiamine-fortified salt for future use in Cambodia (ideal thiamine dose, usual salt intake), and to explore the impact of various doses of maternal thiamine on markers of infant cognitive development.

Study outcomes and analysis objectives

Primary Outcome and Analysis Objective: To estimate the dose on the dose response curve where additional maternal intake of thiamine (oral dose) no longer meaningfully increases human milk total thiamine concentration at 24 weeks postpartum.

For peer review only

Secondary Outcomes and Analysis Objectives:

1. To estimate the dose on the dose response curve where additional maternal intake of thiamine (oral dose) no longer meaningfully increases infant thiamine diphosphate concentrations (ThDP) 24 weeks postnatally, and assess whether this depends on the presence/absence of a genetic hemoglobin disorder.
2. To estimate the dose on the dose response curve where additional maternal intake of thiamine (oral dose) no longer meaningfully increases human milk total thiamine concentration at 4 and 12 weeks postpartum.
3. To estimate the dose on the dose response curve where additional maternal intake of thiamine (oral dose) no longer meaningfully increases infant transketolase activity at 24 weeks postnatally, and assess whether this depends on the presence/absence of a genetic hemoglobin disorder.
4. To estimate the dose on the dose response curve where additional maternal intake of thiamine (oral dose) no longer meaningfully increases maternal ThDP at 24 weeks postpartum, and assess whether this depends on the presence/absence of a genetic hemoglobin disorder.
5. To estimate the dose on the dose response curve where additional maternal intake of thiamine (oral dose) no longer meaningfully increases maternal transketolase activity at 24 weeks postpartum, and assess whether this depends on the presence/absence of a genetic hemoglobin disorder.

6. To test for differences between the 4 randomized groups on human milk total thiamine at 4, 12, and 24 weeks postpartum.
7. To test for differences between the 4 randomized groups on maternal ThDP at 24 weeks postpartum, and assess whether this depends on the presence/absence of genetic hemoglobin disorder.
8. To test for differences between the 4 randomized groups on maternal transketolase activity at 24 weeks postpartum and assess whether this depends on the presence/absence of a genetic hemoglobin disorder.
9. To estimate usual household salt intake from mean fortnightly salt disappearance (weight lost, in g).
10. To estimate salt intake among a subset of 100 lactating women, their male partners (if applicable), and their children 24-59 months (if applicable) using observed weighed salt intake records.
11. To estimate sodium intake using 24 hr urinary sodium concentrations among a subset of 100 lactating women.
12. To test for differences between the 0 & 10 mg randomized groups on Composite Mullen and the 5 subscales of the Mullen at 24 weeks postnatally.
13. To test for differences between the 0 & 10 mg randomized groups on Visual Paired Comparison Novelty Score and the attention and processing speed subscales at 24 weeks postnatally.
14. To test for differences between the 0 & 10 mg randomized groups on the Language Preference Task Score at 24 weeks postnatally.

For peer review only

15. To test for differences between 0 & 10 mg randomized groups on oculomotor scores at 24 weeks postnatally.
16. To determine the effect of inflammation, as measured by C-reactive protein (CRP) and α-1-acid-glycoprotein (AGP) on maternal ThDP at 2 and 24 weeks postpartum, and infant ThDP at 24 weeks postnatal.

Methods

Study design and setting

This is a double-blind, four-parallel arm randomized controlled trial. The study timeline and data collection schedule can be found in **Figure 1**. The study is community-based, with data collection taking place in women's homes in Kampong Thom province in central Cambodia. All women live rurally, in villages in the catchment areas of the following health centres: Tboung Kapoeur, Kampong Svay, Sankor, Chey, Salavisai, Prey Kuy, Prey Pros, and Srayov.

Eligibility criteria

All women must provide written informed consent to participate. Participants must meet the following criteria:

- mothers of a newborn;
- aged 18-45 years;
- most recent pregnancy was normal (i.e. no known chronic conditions, preeclampsia, gestational diabetes etc.), and the singleton infant was born without complications (e.g. low birth weight (<2.5 kg), tongue tie, cleft palate);
- intends to exclusively breastfeed for six months;

For peer review only

- resides in Kampong Thom province, Cambodia, and is not planning to move in the next six months;
- is not currently taking, and hasn't taken any thiamine-containing supplements over the previous 4 months;
- is not currently participating in any nutrition programs beyond normal care;
- is willing to consume one capsule daily from 2 weeks through 24 weeks postpartum;
- is willing for her entire household consume only salt provided by the study team; and
- is willing for the following biological samples to be collected: a maternal venous blood sample and human milk sample at 2 weeks postpartum, a human milk sample at 4 and 12 weeks postpartum, and maternal and infant blood samples and a human milk sample at 24 weeks postpartum.

Recruitment, allocation, randomization, and blinding

A participant flow diagram is shown in **Figure 2**. Participants will be recruited through antenatal care visits, as well as through consultation with local village chiefs, elders, and health centre staff. Pregnant women will be advised and provided with a general overview of the research study, and then the names and contact information of interested women will be shared with the research assistants, who follow up after delivery to screen for eligibility, obtain consent, and enroll women. Recruitment will continue on a rolling basis until 320 women are enrolled.

Women will be randomly assigned to one of the four treatment groups as described in **Table 1**. Participants, research assistants, study investigators, and data analysts will be blinded to the randomized groups. The randomization will be stratified by health centre and use randomly

For peer review only

permuted blocks of size 8 within strata to assign participants to one of eight treatment codes in the ratio 1:1:1:1:1:1:1:1 (2 treatment codes per treatment group to assist with blinding). A computer-generated randomization schedule was prepared by the study statisticians using ralloc.ado in Stata (College Station, TX, USA). An independent scientist determined which treatment code corresponded to which treatment group. When a participant is enrolled in the study, the research assistants open an envelope labeled with the study ID that contains the study ID and accompanying treatment code that will be assigned to that participant.

Table 1: Treatment arms for the *Trial of thiamine supplementation in Cambodia.*

Treatment Arm	Thiamine Dose	Rationale
Negative Control	0 mg/day	Negative Control (placebo)
EAR	1.2 mg/day	1 x thiamine EAR for lactating women [19]
Double EAR	2.4 mg/day	2 x thiamine EAR for lactating women [19]
Positive Control	10 mg/day	Positive Control (dose currently given in supplemental form in Myanmar) [4]

EAR, estimated average requirement

Intervention

Women will be asked to consume one capsule daily between 2 and 24 weeks postnatal. The intervention is opaque capsules containing varying amounts of thiamine hydrochloride and cellulose filler, as indicated in **Table 1**. All thiamine is delivered as thiamine hydrochloride, calculated using a 1.271 correction factor (ratio of molecular weights of thiamine hydrochloride and thiamine). Capsules were formulated, compounded, and packaged as 14-day blister packs at the Quinpool Wellness Centre in Halifax, Nova Scotia, Canada.

All capsules were formulated and packed in two batches in July and November 2018. Thiamine content was assessed by an independent lab (USANA, Salt Lake City, UT, USA) before

For peer review only - http://bmjopen.bmj.com/site/about/guidelines.xhtml

distribution to participants, and will continue to be assessed bi-monthly for the duration of the study. Capsules will be deemed acceptable if the average thiamine concentration for each code (n=10 samples per code) falls within ±15% of the values shown in **Table 1**.

Compliance

Compliance will be assessed fortnightly: research assistants will visit the participant's home to collect the old blister pack and complete a capsule count, and to deliver a new blister pack. A woman will be considered compliant if she consumes ≥80% capsules over the 22-week intervention.

Participant and public involvement in research

This research is being conducted in collaboration with the Cambodian Ministry of Health's National Maternal and Child Nutrition Programme, the Cambodian Ministry of Planning's Sub-Committee for Food Fortification, and Kampong Thom province's Provincial Health Department and Health Operational District. Participants and the public will be involved in the dissemination of study results (public village meetings to share findings), however were not involved in research question development, nor the design of the study or intervention.

Data and biological sample collection

Data and biological samples will be collected at enrolment (between t=0 to 2 weeks postpartum), at 2, 4, 12, and 24 weeks postnatal, as well as during fortnightly monitoring visits (see **Figure 1**).

For peer review only

Chart review

Upon enrolment, a healthcare worker in the Health Centre will record data from the dyad's chart such as any interventions/events at birth, maternal age, number of antenatal care visits, number of iron-folic acid tablets consumed, time and date of birth, sex of infant, and infant anthropometrics.

Questionnaires

Using an interviewer-administered questionnaire, we will collect demographic and socioeconomic information, health information including questions about sleep (adapted from [32]) and postpartum depression [33], limited dietary intake data (e.g. perceptions of salt intake, postpartum food taboos [34]), and infant and young child feeding (IYCF) knowledge and behaviors at 2, 12, and 24 weeks postnatal.

Anthropometry

Anthropometric measures are as follows: infants: length, weight, and head circumference; mothers: height and weight. Initial measurements will be taken in the Health Centre at delivery, and all other measurements will be collected in participant's homes (2, 12, and 24 weeks postnatal) using calibrated instruments and standard protocols as per [35].

Fortnightly monitoring

Every two weeks research assistants will visit the participant's home to distribute a new blister pack and assess intervention compliance. They will also administer a short questionnaire about

For peer review only

selling or sharing of salt, IYCF practices, and the household members eating from the common household pot.

Salt disappearance

At the initial home visit after randomization, research assistants will check that the household has removed all salt from the home, and will distribute table salt in specialized study containers. Using calibrated scales (1 g graduation), research assistants will log the initial weight of the salt container(s). Participants will be asked to consume only salt provided, and since Cambodian families eat from a common pot, they will be instructed for all family meals to be prepared using this salt. Salt disappearance will continue to be assessed at fortnightly visits.

We will also collect supplemental information in a subset of households (n=100) using observed weighed intakes and maternal urinary sodium concentrations (randomly selected from larger study, with a second day repeat on non-consecutive days within 1 week of initial visit). The participant (mother), the husband/man in household aged 18-50 years, and a child between 24-59 months (husband/man and child will not be applicable in all households) will be enrolled in this sub-study. Research assistants will sit in the participants home from dawn through dusk, recording, at the individual level, intake for the 1-3 individuals for all table salt and salt-containing condiments (e.g. fish and soy sauces) consumed. For the mothers only, we will assess 24-hour urinary sodium concentrations [25]. The mother will discard her first urine upon awakening, record the time, and then collect all urine over the next 24 hours into a provided container; the collection will end after the woman's first void the following morning. The research assistants will visit her home to weigh the full container and then take aliquots for

For peer review only

For peer review only - http://bmjopen.bmj.com/site/about/guidelines.xhtml

testing. The urine sample will be temporarily stored at -20°C in Kampong Thom for <2 weeks before being transported to the National Institute for Public Health Laboratory (NIPHL) in Phnom Penh for urinary sodium analysis on a EasyLtye Na/K/Cl Analyser (Medica, Dusseldorf, Germany) and urinary creatinine assessment using a Kenza 240TX Analyser (Biolabo Diagnostics, Maizy, France). We will model thiamine fortification of salt using the University of Iowa's Intake Monitoring, Assessment and Planning Program software (http://www.side.stat.iastate.edu/imapp.php).

Venous blood samples

Trained, Khmer-speaking nurses will meet mothers and infants at their home or a central village location (health centre, or village chief's home) to collect maternal and infant blood samples into EDTA-coated tubes. Maternal blood samples (9 mL) will be collected at 2 and 24 weeks postnatal; infant samples (5 mL) will be collected only at 24 weeks postnatal. Time of day and time since last meal will be recorded.

Human milk samples

Human milk samples will be collected using a battery-powered single breast pump (Swing Breast pump, Medela) at 2, 4, 12, and 24 weeks postnatal. One full breast expression (single breast) will be collected from the breast women self-identify as being more 'full' (the breast not most recently emptied). Since time of day has little effect on milk thiamine concentrations [36], samples can be collected at any time of day, however, time of day, time since last meal, and breast side will be recorded.

For peer review only

Blood and human milk processing

Biological samples will be collected in the village, placed on ice, and transported to the field lab in Kampong Thom within 5 hours of collection for processing. All samples will be stored at -20°C for <2 weeks before being transported the NIPHL for storage at -80°C. Samples will be batch-shipped on dry ice after the 24 weeks postnatal data collection is completed.

Blood and human milk analysis

Venous blood samples will undergo analysis for both transketolase activity and thiamine diphosphate concentrations (ThDP) at the NIHR BRC Nutritional Biomarker Laboratory at the University of Cambridge in the United Kingdom. At 2 weeks postnatal, maternal whole blood ThDP and erythrocyte transketolase activity will be measured. At 24 weeks postnatal, ThDP and transketolase activity will be measured in both whole blood and erythrocytes, and infant samples will be assayed for whole blood ThDP and erythrocyte transketolase activity. When ThDP is assessed in whole blood, concentrations must be normalized to hemoglobin concentrations and/or hematocrit [37] measured via a HemoCue 201 portable hemoglobinometer and capillary hematocrit tubes, respectively.

Evidence from the 2014 Cambodian National Micronutrient survey indicates that eThDP is affected by the presence/absence of genetic hemoglobin disorders (unpublished data). With this, it is vital to assess genetic hemoglobin disorders, as this could influence participant’s response to thiamine, it may influence transketolase, and it could change thiamine dosage requirements in other countries. Therefore, at one timepoint only (2 weeks postnatal for maternal, and 24 weeks postnatal for infant samples), samples will undergo hemoglobin capillary electrophoresis

For peer review only

analysis to identify structural hemoglobin variants [38]. Buffy coat samples will be stored in the BioBank in the Department of Applied Human Nutrition at Mount Saint Vincent University in Halifax, Nova Scotia, Canada, for potential later assessment of genetic hemoglobin disorders.

Biomarkers may be influenced by inflammation, which is common in this population [39,40]. Plasma samples will be sent to Dr. Jurgen Erhardt at the VitMin Lab in Germany for analysis of C-reactive protein (CRP) and α-1-acid-glycoprotein (AGP) using an immunosorbent assay [41]. Note that this assay will also measure retinol binding protein (RBP), ferritin, and soluble transferrin receptor (sTfR). Other plasma samples will be stored in the BioBank for potential future use.

The weight of the full human milk expression will be recorded, the sample mixed, and 2 mL aliquots obtained in amber cryovials. Human milk thiamine concentrations will be measured at the USDA/ARS Western Human Nutrition Research Center, University of California, Davis [42]. Additional human milk samples will be stored in the BioBank for potential future use.

Cognitive assessments

We will conduct cognitive assessments at 2, 12, and 24 weeks postnatal; see **Figure 1** for battery of assessments at each timepoint. Assessments include: the Hammersmith Infant Neurological Exam, Infant Mullen Scales of Early Learning, a Primary Engagement Task, a Secondary Engagement Task, the Caregiver Reported Early Development Instruments (CREDI), an oculomotor exam, the Visual Paired Comparison task, and the Language Preference task.

For peer review only

The Hammersmith Infant Neurological Exam is a standard tool for providing basic information about infants' neurological status through use of gentle touch and gentle social interaction in order to examine infants' sensory and motor responses; it has previously shown excellent interobserver reliability even among less experienced staff [43]. The Infant Mullen Scales of Early Learning is an individually administered, multi-domain measure of early development, with scales measuring development in visual reception, fine motor, gross motor, receptive language, and expressive language [44]. The Primary Engagement Task measures individual variation in infant, caregiver, and dyad-level developmental change in the ability to engage contingently with the partner in direct positive mutual engagement interactions. The Secondary Engagement Task measures individual variation in caregiver and infant ability to engage mutually in relation to an external object. The CREDI is a caregiver-reported measure of child development that has been shown to have right validity and reliability in low-resource settings [45]. An oculomotor exam has been included because oculumotor disturbances are not uncommon among thiamine deficient adults presenting with Wernicke's encephalopathy [46]. The Visual Paired Comparison task probes infants' recognition memory and attention, and has been used to predict subsequent verbal IQ in middle childhood [47,48], and produce measures of attention predictive of developmental maturity and better cognitive function [49,50]. The Language Preference Task is designed to measure individual variation in infants' level of interest in the kind of language that caregivers typically direct toward infants (aka infant-directed talk or 'motherese') relative to a) the kind of language that is typically directed toward adults (aka adult-directed talk), and b) sounds that are matched in complexity but are non-linguistic (non-linguistic analog). Over a series of trials, infants hear these three different kinds of sound samples through an audio speaker (infant-directed talk, adult-directed talk, non-linguistic analog), and the

For peer review only

duration that they look toward a visual display during each sound sample is subsequently measured from the videotaped record collected during the session.

Participant remuneration

All participants will receive a mobile phone and mobile phone credits. In addition, modest, study-appropriate remuneration such as a sarong, canned fish, or laundry soap will be provided at biological sample collection points (2, 4, 12, and 24 weeks postnatal, and after urine collection).

Safety considerations, safety monitoring, and breaking the blinding

There is no UL for thiamine [19,25,26], and therefore no necessity for a Serious Adverse Events Committee or a Data Safety Monitoring Board in the current study. While we are confident that there is a very low risk of serious adverse events in this study related to the intervention, we do expect some infant deaths in our cohort due to other causes. Per the 2014 Cambodian Demographic and Health Survey rates of neonatal (birth to one month) and infant (birth to one year) mortality [51], we calculated an expected mortality rate in our study to lie between 1.67 and 3.68 infant deaths.

The master ID list will not be unblinded during the study unless in the unlikely event of an adverse event in the trial. If there is a medical emergency and unblinding of a participant is required, our blinding mechanism would allow for only one of the two codes per treatment arm to be revealed (as each treatment arm will have two codes). We would then document and report to the funding agency and all ethics committees why any premature unblinding occurred.

For peer review only

Once data collection and cleaning is complete, the database will be locked and unblinded treatment codes will be included in the database because a blinded analysis is not possible for estimating dose response curves.

Sample size calculations

To detect a clinically meaningful difference of 40 μg/L in human milk total thiamine concentration between any two treatment groups with 90% power, assuming a SD of 43 μg/L (estimated SD of control group in a Cambodian thiamine-fortified fish sauce trial [52]), 48 women are required per treatment group, or a total of 192 women. This sample size allows for 20% attrition and uses a two-sided alpha of 0.0083 for each of the 6 pairwise comparisons between the four treatment groups in order to control the familywise error rate at the 0.05 level using a Bonferroni adjustment for multiple comparisons. Recruitment of 320 participants (80 per group) was planned to allow for some uncertainty in the assumed values, particularly the SD which may be larger than anticipated.

Simulations of 500 dose-response curves for human milk total thiamine concentration were conducted to estimate the precision that this sample size would provide for addressing the primary study objective (i.e. for estimating the dose on the dose response curve where additional maternal intake of thiamine (oral dose) no longer meaningfully increases human milk total thiamine concentration at 24 weeks postpartum, defined as the dose required to achieve 90% of the average maximum human milk total thiamine concentration). Data were simulated based on an average minimum and maximum concentration of 136 μg/L [52] and 210 μg/L [19], respectively, with a SD of 43 μg/L [52], and reaching 50% and 90% of the maximum average

For peer review only

concentration at dose 1.2 mg/d (EAR group) and 2.4 mg/d (double EAR group) respectively. Assuming an E_{max} dose-response curve, the precision of the estimated dose is ±1.29 mg/d (i.e. the 95% confidence interval for the estimated dose will be within ±1.29 mg/d of the point estimate).

Data and sample management and security

Participants will be given a unique alpha-numeric study ID code, not derived from personal identifiers; this code will link all data collected from this individual. All data will be collected directly on tablets using structured forms designed specifically for this study. Data will be reviewed daily by the research assistant and field supervisor before secure download and integration with the larger database. Data management and security procedures, including assurance of confidentiality, adhere to the Canadian Tri-Council Policy Statement on Ethical Conduct for Research Involving Humans (TCPS2 CORE) guidelines, and are outlined in full in the protocol at clinicaltrials.gov (NCT03616288).

Data analysis plan

A full statistical analysis plan is published in the clinicaltrials.gov entry (NCT03616288). The primary analyses will be performed on an 'intention to treat' basis, where all participants will be analyzed according to their allocated treatment group regardless of compliance. A secondary 'per-protocol' analysis will be performed on the subset of women who consumed ≥80% capsules over the study period. The parameters of the E_{max} dose-response curves will be estimated using non-linear least squares models. Separate curves will be fitted for outcomes measured at 4 weeks, 12 weeks, and 24 weeks postnatal. The dose where additional maternal intake of thiamine

For peer review only

467 no longer meaningfully increases milk thiamine concentration, defined as the dose that achieves
468 90% of the maximum concentration, will be estimated from the fitted curve with standard errors
469 estimated by bootstrapping for calculating 95% confidence intervals.
470
471 Comparisons between treatment groups will be performed using linear mixed-effects models to
472 account for repeated measurements and adjusted for randomization strata and levels at 2 weeks
473 postnatal. Interaction tests will be performed to assess whether treatment effects depend on
474 genetic hemoglobin disorder. Missing data will be addressed using multiple imputation to create
475 100 complete datasets for analysis, with a sensitivity analysis performed on the raw (unimputed)
476 data [53].
477
478
479
480 *Ethics and dissemination*
481 Approvals were obtained from the following Research Ethics Boards: the National Ethics
482 Committee for Health Research, Cambodia (112/250NECHR), Mount Saint Vincent University
483 Research Ethics Committee, Canada (2017-141); and the University of Oregon Institutional
484 Review Board, USA (07052018.008). This study is registered at clinicaltrials.gov
485 (ClinicalTrials.gov Identifier NCT03616288 [August 7, 2018]; Protocol version 2.1 [July 24,
486 2018]).
487
488 Updates on the study will be shared at the monthly Cambodian Nutrition Working Group
489 meetings, attended by researchers and non-governmental organizations (NGO) engaging in

For peer review only

nutrition research and programming in Cambodia. Scaling Up Nutrition and other relevant groups will also be updated regularly. Research results will be presented at academic nutrition, public health, and psychology conferences, and in peer-reviewed journals that offer open access. Data will be made available on a public repository after dissemination.

We plan to create various lay outputs from this study that can be used by NGO and government agencies working with families, or can be accessed directly by families. We will host a Dissemination Workshop in Phnom Penh open to relevant stakeholders (NGOs, government, researchers, media, clinicians, public health, all sectors) to share the main outcomes of the study, and officially 'launch' lay resources. We will also return to each community in Kampong Thom to conduct a village-wide meeting relaying study results.

Trial status

Participant recruitment began in August 2018, and data collection started September 12, 2018. We estimate data collection will be completed by June 2019.

Discussion

Suboptimal maternal thiamine intake and/or status puts exclusively breastfed infants at risk of low thiamine status [12], impaired cognitive development [15–17], and infantile beriberi, which can be fatal [13]. Thiamine fortification of salt is a potentially low-cost and sustainable means of combating suboptimal thiamine status, however knowledge gaps must be filled before thiamine

For peer review only

fortification can proceed [4]. There are limited data available on the dose of thiamine required by lactating women to optimize the thiamine concentrations in their milk. Further, usual salt intake among lactating women is unknown. Finally, although there is emerging evidence that low thiamine intake in early life impacts cognitive development, this has yet to be assessed in a controlled research environment. This study is posed to address these knowledge gaps and provide valuable information to inform any potential future thiamine fortification efforts in Cambodia or bordering countries.

Acknowledgements

We thank the members of the study's Scientific Advisory Board for valuable input on the study protocol (alphabetical order): Drs. Megan Bourassa, Levente Diosady, Lisa Houghton, Arnaud Laillou, and Annie Wesley. We thank Dr. Ken Brown for assistance and insight during study conception, Dr. Geraldine Richmond for her role in early protocol development, and Keith Porter for early input on project feasibility and implementation. We acknowledge USANA (Salt Lake City, UT, USA) for the generous provision of laboratory analysis for study capsule thiamine hydrochloride content.

Author's Contributions

KCW drafted the manuscript. KCW, HK, TJG, FW, JRM, and DB conceived the study and wrote the initial study protocol. MB, SP, LY, SL, KC, & JG assisted in developing the protocol. HK, MB, & PS facilitated implementation of the study. LY & SL developed the statistical analysis plan. KC & JG are involved in study coordination. All authors participated in, read, and approved the final manuscript.

For peer review only

Funding

This study is funded through the Bill & Melinda Gates Foundation and the Sackler Institute for Nutrition Science, New York Academy of Sciences (Opportunity ID OPP1176128) as 'Objective 3: Trial of thiamine supplementation in Cambodia', as part of the larger program grant entitled 'Improving estimates of the global burden of thiamine deficiency disorders (TDDs) and approaches to their control'. The study funders were involved in early study conception; funders will have no involvement in the study design, collection, management, analysis, and interpretation of data, writing of the report, nor the decision to submit the report for publication. LNY was supported by an Australian National Health and Medical Research Council Early Career Fellowship (ID 1052388).

Completing Interest Statement

None to declare.

List of Figures

Figure 1: Study timeline and data collection schedule for *Trial of thiamine supplementation in Cambodia.*

Figure 1 legend. CREDI: Caregiver Reported Early Development Instruments; wk: week.

Figure 2: Participant flow chart for *Trial of thiamine supplementation in Cambodia.*

Figure 2 legend. Double EAR: Double EAR group (2.4 mg thiamine as thiamine hydrochloride); EAR: EAR group (1.2 mg thiamine as thiamine hydrochloride); Placebo:

For peer review only

559 negative control group (placebo; 0 mg thiamine); Positive Control: Positive Control group (10
560 mg thiamine as thiamine hydrochloride).
561
562

For peer review only

References

1 Bemeur C, Butterworth RF. Thiamin. In: Ross AC, Calallero B, Cousins RJ, *et al.*, eds. *Modern Nutrition in Health and Disease*. Baltimore, MD: Lippincott Williams & Wilkins 2014. 317–24.

2 Fattal-Valevski A. Thiamine (Vitamin B1). *J Evid Based Complementary Altern Med* 2011;**16**:12–20. doi:10.1177/1533210110392941

3 Khounnorath S, Chamberlain K, Taylor AM, *et al.* Clinically unapparent infantile thiamin deficiency in Vientiane, Laos. *PLoS Negl Trop Dis* 2011;**5**:e969. doi:10.1371/journal.pntd.0000969

4 Whitfield KC, Bourassa MW, Adamolekun B, *et al.* Thiamine deficiency disorders: diagnosis , prevalence , and a roadmap for global control programs. *Ann N Y Acad Sci* 2018;**1430**:3–43. doi:10.1111/nyas.13919

5 Whitfield KC, Karakochuk CD, Liu Y, *et al.* Poor thiamin and riboflavin status is common among women of childbearing age in rural and urban Cambodia. *J Nutr* 2015;**145**. doi:10.3945/jn.114.203604

6 Whitfield KC, Green TJ. Erratum for Whitfield et al. Poor thiamin status is common among women of childbearing age in rural and urban Cambodia. J Nutr 2015;145:628-33. *J Nutr* 2016;**146**:147–8. doi:10.3945/jn.115.225599.147

7 Coats D, Shelton-Dodge K, Ou K, *et al.* Thiamine deficiency in Cambodian infants with and without beriberi. *J Pediatr* 2012;**161**:843–7.

8 Whitfield KC, Karakochuk CD, Kroeun H, *et al.* Perinatal consumption of thiamine-fortified fish sauce in rural Cambodia: A randomized clinical trial. *JAMA Pediatr* 2016;**170**. doi:10.1001/jamapediatrics.2016.2065

9 Keating EM, Nget P, Kea S, *et al.* Thiamine deficiency in tachypnoeic Cambodian infants. *Paediatr Int Child Health* 2014;**35**:312–8. doi:10.1179/2046905514Y.0000000162

10 Porter SG, Coats D, Fischer PR, *et al.* Thiamine deficiency and cardiac dysfunction in Cambodian infants. *J Pediatr* 2014;**164**:1456–61. doi:10.1016/j.jpeds.2014.01.049

11 Whitfield KC, Smith G, Chamnan C, *et al.* High prevalence of thiamine (vitamin B1) deficiency in early childhood among a nationally representative sample of Cambodian women of childbearing age and their children. *PLoS Negl Trop Dis* 2017;**11**. doi:10.1371/journal.pntd.0005814

12 Allen LH. B vitamins in breast milk: relative importance of maternal status and intake, and effects on infant status and function. *Adv Nutr* 2012;**3**:362–9.

13 Luxemburger C, White NJ, ter Kuile F, *et al.* Beri-beri: the major cause of infant mortality in Karen refugees. *Trans R Soc Trop Med Hyg* 2003;**97**:251–5.

14 Fattal-Valevski A, Kesler A, Sela B-A, *et al.* Outbreak of life-threatening thiamine deficiency in infants in Israel caused by a defective soy-based formula. *Pediatrics* 2005;**115**:233–8. doi:10.1542/peds.2004-1255

15 Harel Y, Zuk L, Guindy M, *et al.* The effect of subclinical infantile thiamine deficiency on motor function in preschool children. *Matern Child Nutr* 2017;e12397. doi:10.1111/mcn.12397

16 Fattal-Valevski A, Azouri-Fattal I, Greenstein YJ, *et al.* Delayed language development due to infantile thiamine deficiency. *Dev Med Child Neurol* 2009;**51**:629–34. doi:10.1111/j.1469-8749.2008.03161.x

17 Fattal I, Friedmann N, Fattal-Valevski A. The crucial role of thiamine in the development of syntax and lexical retrieval: a study of infantile thiamine deficiency. *Brain*

For peer review only

2011;**134**:1720–39. doi:10.1093/brain/awr068
18 Prado EL, Dewey KG. Nutrition and brain development in early life. *Nutr Rev* 2014;**72**:267–84. doi:10.1111/nure.12102
19 Institute of Medicine. Thiamin. In: *Dietary Reference Intakes for thiamin, riboflavin, niacin, vitamin B6, folate, vitamin B12, pantothenoc acid, biotin, and choline*. Washington, DC: : National Academy Press 1998. 58–86.
20 FAO Statistics Division. Food Balance Sheets: Cambodia (2011). 2014. http://faostat.fao.org/site/368/DesktopDefault.aspx?PageID=368#ancor
21 Allen LH, de Benoist B, Dary O, *et al. Guidelines on food fortification with micronutrients*. Geneva, Switzerland: : World Health Organization and Food and Agriculture Organization of the United Nations 2006.
22 Dwyer JT, Wiemer KL, Dary O, *et al.* Fortification and health: challenges and opportunities. *Adv Nutr* 2015;**6**:124–31. doi:10.3945/an.114.007443.micronutrient
23 Smith G. Micronutrient fortification of food: issues for Asia. *J Nutr Sci Vitaminol (Tokyo)* 2015;**61**:S183-5.
24 Hurrell R. *The Mineral Fortification of Foods*. Surrey, UK: Leatherhead Publishing 1999.
25 Gibson RS. *Principles of Nutritional Assessment*. 2nd ed. New York, NY, USA: Oxford University Press 2005.
26 Smithline HA, Donnino M, Greenblatt DJ. Pharmacokinetics of high-dose oral thiamine hydrochloride in healthy subjects. *BMC Clin Pharmacol* 2012;**12**:4. doi:10.1186/1472-6904-12-4
27 Whitfield KC, Karakochuk CD, Kroeun H, *et al.* Perinatal consumption of thiamine-fortified fish sauce in rural Cambodia: a randomized clinical efficacy trial. *JAMA Pediatr* 2016;**170**:e162065. doi:10.1001/jamapediatrics.2016.2065
28 Nilles EJ, Manaia A, Ruaia B, *et al.* Re-emergence of thiamine deficiency disease in the Pacific islands (2014 – 15): a case-control study. *PLoS One* 2018;**13**:e0198590.
29 Adamolekun B, Hiffler L. A diagnosis and treatment gap for thiamine deficiency disorders in sub-Saharan Africa? *Ann N Y Acad Sci* 2017;**1408**:15–9. doi:10.1111/nyas.13509
30 United Nations Children's Fund (UNICEF). *Improving child nutrition: the achievable imperative for global progress*. New York, USA: 2013. doi:978-92-806-4686-3
31 Andersson M, Takkouche B, Egli I, *et al.* Current global iodine status and progress over the last decade towards the elimination of iodine deficiency. *Bull World Health Organ* 2005;**83**:518–25.
32 Keller MA, Goldbery WA. Co-sleeping: help or hinderance for young children's independence? *Infant Child Dev* 2004;**13**:369–88. doi:10.1002/icd.
33 Gjerdingen D, Crow S, McGovern P, *et al.* Postpartum depression screening at well-child visits: validity of a 2-question screen and the PHQ-9. *Ann Fam Med* 2009;**7**:63–70. doi:10.1370/afm.933.Department
34 Wallace LJ, Summerlee AJS, Dewey CE, *et al.* Women's nutrient intakes and food-related knowledge in rural Kandal province, Cambodia. *Asia Pac J Clin Nutr* 2014;**23**:263–71. doi:10.6133/apjcn.2014.23.2.02
35 Cogill B. Anthropometric Indicators Measurement Guide. Washington, DC: 2003.
36 Hampel D, Shahab-Ferdows S, Islam MM, *et al.* Vitamin concentrations in human milk vary with time within feed, circadian rhythym, and single-dose supplementation. *J Nutr* 2017;**147**:603–11. doi:10.3945/jn.116.242941.1
37 Floridi A, Pupita A, Palmerini C, *et al.* Thiamine pyrophosphate determination in whole

For peer review only

blood and erythrocytes by high performance liquid chromatography. *J Int Vitaminol Nutr* 1983;**54**:165–171.

38 Hartwell SK, Srisawang B, Kongtawelert P, *et al.* Review on screening and analysis techniques for hemoglobin variants and thalassemia. *Talanta* 2005;**65**:1149–61. doi:10.1016/j.talanta.2004.09.013

39 Makurat J, Friedrich H, Kuong K, *et al.* Nutritional and micronutrient status of female workers in a garment factory in Cambodia. *Nutrients* 2016;**8**. doi:10.3390/nu8110694

40 Yong T-S, Chai J-Y, Sohn W-M, *et al.* Prevalence of intestinal helminths among inhabitants of Cambodia (2006-2011). *Korean J Parasitol* 2014;**52**:661–6.

41 Erhardt JG, Estes JE, Pfeiffer CM, *et al.* Combined measurement of ferritin, soluble transferrin receptor, retinol binding protein, and C-reactive protein by an inexpensive, sensitive, and simple sandwich enzyme-linked immunosorbent assay technique. *J Nutr* 2004;**134**:3127–32. doi:134/11/3127 [pii]

42 Hampel D, York ER, Allen LH. Ultra-performance liquid chromatography tandem mass-spectrometry (UPLC-MS/MS) for the rapid, simultaneous analysis of thiamin, riboflavin, flavin adenine dinucleotide, nicotinamide and pyridoxal in human milk. *J Chromatogr B* 2012;**903**:7–13. doi:10.1016/j.jchromb.2012.06.024

43 Maitre NL, Chorna O, Romeo DM, *et al.* Implementation of the Hammersmith Infant Neurological Examination in a high-risk infant follow-up program. *Pediatr Neurol* 2016;**65**:31–8. doi:10.1016/j.pediatrneurol.2016.09.010

44 Mullen EM. Mullen scales of early learning. 1995.

45 McCoy DC, Sudfeld CR, Bellinger DC, *et al.* Development and validation of an early childhood development scale for use in low-resourced settings. *Popul Health Metr* 2017;**15**.

46 Sechi G, Serra A. Wernicke's encephalopathy: new clinical settings and recent advances in diagnosis and management. *Lancet Neurol* 2007;**6**:442–55. doi:10.1016/S1474-4422(07)70104-7

47 McCall RB. What process mediates predictions of childhood IQ from infant habituation and recognition memory? Speculations on the roles of inhibition and rate of information processing. *Intelligence* 1994;**18**:107–25. doi:10.1016/0160-2896(94)90022-1

48 Sigman M, Cohen SE, Beckwith L. Why does infant attention predict adolescent intelligence? *Infant Behav Dev* 1997;**20**:133–40. doi:10.1016/S0163-6383(97)90016-3

49 Rose SA, Feldman JF, Jankowski JJ. Dimensions of cognition in infancy. *Intelligence* 2004;**32**:245–62. doi:10.1016/j.intell.2004.01.004

50 Rose SA, Feldman JF, Jankowski JJ, *et al.* A cognitive cascade in infancy: Pathways from prematurity to later mental development. *Intelligence* 2008;**36**:367–78. doi:10.1016/j.intell.2007.07.003

51 National Institute of Statistics, Directorate General for Health, ICF International. Cambodia Demographic and Health Survey 2014. Phnom Penh, Cambodia and Rockville, Maryland, USA: 2015.

52 Whitfield KC, Karakochuk CD, Kroeun H, *et al.* Household Consumption of Thiamin-Fortified Fish Sauce Increases Erythrocyte Thiamin Concentrations among Rural Cambodian Women and Their Children Younger Than 5 Years of Age: A Randomized Controlled Efficacy Trial. *J Pediatr* 2017;**181**. doi:10.1016/j.jpeds.2016.10.066

53 Graham JW, Olchowski AE, Gilreath TD. How many imputations are really needed? Some practical clarifications of multiple imputation theory. *Prev Sci* 2007;**8**:206–13.

For peer review only

doi:10.1007/s11121-007-0070-9

For peer review only

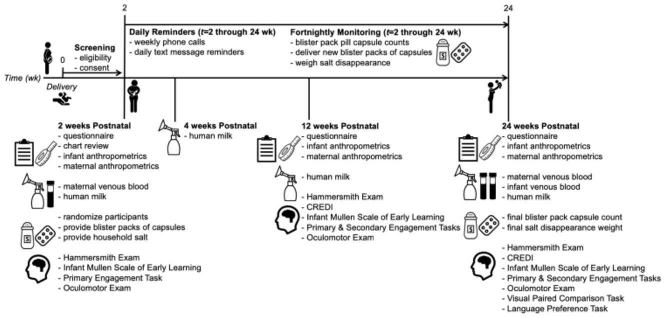

[Caption]: Figure 1: Study timeline and data collection schedule for Trial of thiamine supplementation in Cambodia.
[Legend]: CREDI: Caregiver Reported Early Development Instruments; wk: week.

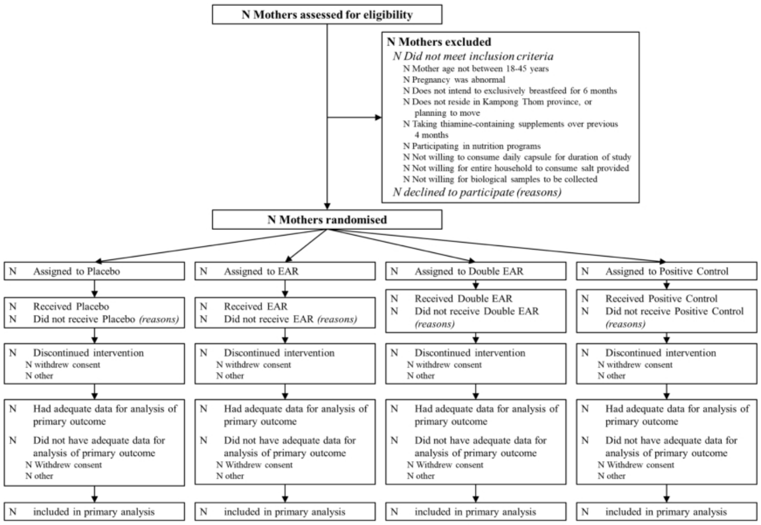

[Caption]: Figure 2: Participant flow chart for Trial of thiamine supplementation in Cambodia.
[Legend]: Double EAR: Double EAR group (2.4 mg thiamine as thiamine hydrochloride); EAR: EAR group (1.2 mg thiamine as thiamine hydrochloride); Placebo: negative control group (placebo; 0 mg thiamine); Positive Control: Positive Control group (10 mg thiamine as thiamine hydrochloride).

STANDARD PROTOCOL ITEMS: RECOMMENDATIONS FOR INTERVENTIONAL TRIALS

SPIRIT 2013 Checklist: Recommended items to address in a clinical trial protocol and related documents*

Section/item	Item No	Description	
**Administrative information**			
Title	1	Descriptive title identifying the study design, population, interventions, and, if applicable, trial acronym	P1
Trial registration	2a	Trial identifier and registry name. If not yet registered, name of intended registry	P2,19
	2b	All items from the World Health Organization Trial Registration Data Set	included
Protocol version	3	Date and version identifier	P20
Funding	4	Sources and types of financial, material, and other support	P22
Roles and responsibilities	5a	Names, affiliations, and roles of protocol contributors	P1,22
	5b	Name and contact information for the trial sponsor	P22
	5c	Role of study sponsor and funders, if any, in study design; collection, management, analysis, and interpretation of data; writing of the report; and the decision to submit the report for publication, including whether they will have ultimate authority over any of these activities	P22
	5d	Composition, roles, and responsibilities of the coordinating centre, steering committee, endpoint adjudication committee, data management team, and other individuals or groups overseeing the trial, if applicable (see Item 21a for data monitoring committee)	P16,17, 22
**Introduction**			
Background and rationale	6a	Description of research question and justification for undertaking the trial, including summary of relevant studies (published and unpublished) examining benefits and harms for each intervention	P3-7
	6b	Explanation for choice of comparators	P9
Objectives	7	Specific objectives or hypotheses	P4-7
Trial design	8	Description of trial design including type of trial (eg, parallel group, crossover, factorial, single group), allocation ratio, and framework (eg, superiority, equivalence, noninferiority, exploratory)	P7

For peer review only

Methods: Participants, interventions, and outcomes

Study setting	9	Description of study settings (eg, community clinic, academic hospital) and list of countries where data will be collected. Reference to where list of study sites can be obtained	P7
Eligibility criteria	10	Inclusion and exclusion criteria for participants. If applicable, eligibility criteria for study centres and individuals who will perform the interventions (eg, surgeons, psychotherapists)	P7-8
Interventions	11a	Interventions for each group with sufficient detail to allow replication, including how and when they will be administered	P9
	11b	Criteria for discontinuing or modifying allocated interventions for a given trial participant (eg, drug dose change in response to harms, participant request, or improving/worsening disease)	P16-17
	11c	Strategies to improve adherence to intervention protocols, and any procedures for monitoring adherence (eg, drug tablet return, laboratory tests)	P10
	11d	Relevant concomitant care and interventions that are permitted or prohibited during the trial	NA
Outcomes	12	Primary, secondary, and other outcomes, including the specific measurement variable (eg, systolic blood pressure), analysis metric (eg, change from baseline, final value, time to event), method of aggregation (eg, median, proportion), and time point for each outcome. Explanation of the clinical relevance of chosen efficacy and harm outcomes is strongly recommended	P6-7
Participant timeline	13	Time schedule of enrolment, interventions (including any run-ins and washouts), assessments, and visits for participants. A schematic diagram is highly recommended (see Figure)	P8-16, Fig2
Sample size	14	Estimated number of participants needed to achieve study objectives and how it was determined, including clinical and statistical assumptions supporting any sample size calculations	P17-18
Recruitment	15	Strategies for achieving adequate participant enrolment to reach target sample size	P8

Methods: Assignment of interventions (for controlled trials)

Allocation:

Sequence generation	16a	Method of generating the allocation sequence (eg, computer-generated random numbers), and list of any factors for stratification. To reduce predictability of a random sequence, details of any planned restriction (eg, blocking) should be provided in a separate document that is unavailable to those who enrol participants or assign interventions	P8-9

For peer review only

Allocation concealment mechanism	16b	Mechanism of implementing the allocation sequence (eg, central telephone; sequentially numbered, opaque, sealed envelopes), describing any steps to conceal the sequence until interventions are assigned	P9
Implementation	16c	Who will generate the allocation sequence, who will enrol participants, and who will assign participants to interventions	P8-9
Blinding (masking)	17a	Who will be blinded after assignment to interventions (eg, trial participants, care providers, outcome assessors, data analysts), and how	P8,17
	17b	If blinded, circumstances under which unblinding is permissible, and procedure for revealing a participant's allocated intervention during the trial	P17
**Methods: Data collection, management, and analysis**			
Data collection methods	18a	Plans for assessment and collection of outcome, baseline, and other trial data, including any related processes to promote data quality (eg, duplicate measurements, training of assessors) and a description of study instruments (eg, questionnaires, laboratory tests) along with their reliability and validity, if known. Reference to where data collection forms can be found, if not in the protocol	P10-16
	18b	Plans to promote participant retention and complete follow-up, including list of any outcome data to be collected for participants who discontinue or deviate from intervention protocols	P11
Data management	19	Plans for data entry, coding, security, and storage, including any related processes to promote data quality (eg, double data entry; range checks for data values). Reference to where details of data management procedures can be found, if not in the protocol	P18-19
Statistical methods	20a	Statistical methods for analysing primary and secondary outcomes. Reference to where other details of the statistical analysis plan can be found, if not in the protocol	P19
	20b	Methods for any additional analyses (eg, subgroup and adjusted analyses)	P19
	20c	Definition of analysis population relating to protocol non-adherence (eg, as randomised analysis), and any statistical methods to handle missing data (eg, multiple imputation)	P19
**Methods: Monitoring**			
Data monitoring	21a	Composition of data monitoring committee (DMC); summary of its role and reporting structure; statement of whether it is independent from the sponsor and competing interests; and reference to where further details about its charter can be found, if not in the protocol. Alternatively, an explanation of why a DMC is not needed	P16-17

For peer review only

	21b	Description of any interim analyses and stopping guidelines, including who will have access to these interim results and make the final decision to terminate the trial	NA
Harms	22	Plans for collecting, assessing, reporting, and managing solicited and spontaneously reported adverse events and other unintended effects of trial interventions or trial conduct	P17
Auditing	23	Frequency and procedures for auditing trial conduct, if any, and whether the process will be independent from investigators and the sponsor	NA
**Ethics and dissemination**			
Research ethics approval	24	Plans for seeking research ethics committee/institutional review board (REC/IRB) approval	P2,20
Protocol amendments	25	Plans for communicating important protocol modifications (eg, changes to eligibility criteria, outcomes, analyses) to relevant parties (eg, investigators, REC/IRBs, trial participants, trial registries, journals, regulators)	P17
Consent or assent	26a	Who will obtain informed consent or assent from potential trial participants or authorised surrogates, and how (see Item 32)	P8
	26b	Additional consent provisions for collection and use of participant data and biological specimens in ancillary studies, if applicable	P8, 14-15
Confidentiality	27	How personal information about potential and enrolled participants will be collected, shared, and maintained in order to protect confidentiality before, during, and after the trial	P18
Declaration of interests	28	Financial and other competing interests for principal investigators for the overall trial and each study site	P22
Access to data	29	Statement of who will have access to the final trial dataset, and disclosure of contractual agreements that limit such access for investigators	P20
Ancillary and post-trial care	30	Provisions, if any, for ancillary and post-trial care, and for compensation to those who suffer harm from trial participation	NA
Dissemination policy	31a	Plans for investigators and sponsor to communicate trial results to participants, healthcare professionals, the public, and other relevant groups (eg, via publication, reporting in results databases, or other data sharing arrangements), including any publication restrictions	P2,20
	31b	Authorship eligibility guidelines and any intended use of professional writers	NA
	31c	Plans, if any, for granting public access to the full protocol, participant-level dataset, and statistical code	P20

For peer review only - http://bmjopen.bmj.com/site/about/guidelines.xhtml

Appendices

Informed consent materials	32	Model consent form and other related documentation given to participants and authorised surrogates	NA
Biological specimens	33	Plans for collection, laboratory evaluation, and storage of biological specimens for genetic or molecular analysis in the current trial and for future use in ancillary studies, if applicable	P12-15

*It is strongly recommended that this checklist be read in conjunction with the SPIRIT 2013 Explanation & Elaboration for important clarification on the items. Amendments to the protocol should be tracked and dated. The SPIRIT checklist is copyrighted by the SPIRIT Group under the Creative Commons "Attribution-NonCommercial-NoDerivs 3.0 Unported" license.

For peer review only

BMJ Open

Thiamine dose response in human milk with supplementation among lactating women in Cambodia: study protocol for a double-blind, four-parallel arm randomized controlled trial

Journal:	*BMJ Open*
Manuscript ID	bmjopen-2019-029255.R1
Article Type:	Protocol
Date Submitted by the Author:	23-Apr-2019
Complete List of Authors:	Whitfield, Kyly; Mount Saint Vincent University<br>Kroeun, Hou; Helen Keller International Cambodia<br>Green, Tim; South Australian Health & Medical Research Institute<br>Wieringa, Frank; Institut de Recherche pour le Développement, IRD/UM/SupAgro, UMR-204 Nutripass<br>Borath, Mam; Cambodia Ministry of Planning, National Sub-Committee for Food Fortification<br>Sophonneary, Prak; Cambodia Ministry of Health, National Nutrition Programme, Maternal and Child Health Centre<br>Measelle, Jeff; University of Oregon, Department of Psychology<br>Baldwin, Dare; University of Oregon, Department of Psychology<br>Yelland, Lisa; South Australian Health & Medical Research Institute; University of Adelaide, School of Public Health<br>Leemaqz, Shalem; South Australian Health & Medical Research Institute<br>Chan, Kathleen; Mount Saint Vincent University<br>Gallant, Jelisa; Mount Saint Vincent University
**Primary Subject Heading**:	Nutrition and metabolism
Secondary Subject Heading:	Global health, Paediatrics, Public health
Keywords:	thiamine (vitamin B1), dose response, human milk, fortification, salt, infant cognitive development

SCHOLARONE™
Manuscripts

Thiamine dose response in human milk with supplementation among lactating women in Cambodia: study protocol for a double-blind, four-parallel arm randomized controlled trial

Kyly C WHITFIELD[1], HOU Kroeun[2], Tim J GREEN[3], Frank WIERINGA[4], MAM Borath[5], SOPHONNEARY Prak[6], Jeff R MEASELLE[7], Dare BALDWIN[7], Lisa N YELLAND[3,8], Shalem LEEMAQZ[3], Kathleen CHAN[1], Jelisa GALLANT[1]

Corresponding Author: Kyly C. Whitfield, Department of Applied Human Nutrition, Mount Saint Vincent University, 166 Bedford Hwy, Halifax, NS B3M 2J6, Canada. kyly.whitfield@msvu.ca, (902) 457-5978.

Author affiliations

1. Department of Applied Human Nutrition, Mount Saint Vincent University, Halifax, Canada
2. Helen Keller International Cambodia, Phnom Penh, Cambodia
3. South Australian Health and Medical Research Institute, Adelaide, South Australia, Australia
4. UMR-204, Institut de recherche pour le développement, UM/IRD/SupAgro, Montpellier, France
5. National Sub-Committee for Food Fortification, Cambodia Ministry of Planning, Phnom Penh, Cambodia
6. National Nutrition Programme, Maternal and Child Health Centre, Cambodia Ministry of Health, Phnom Penh, Cambodia
7. Department of Psychology, University of Oregon, Eugene, Oregon, USA
8. School of Public Health, The University of Adelaide, Adelaide, South Australia, Australia

Word Count: 4,603

Key Words: thiamine (vitamin B1), dose response, human milk, fortification, salt, infant cognitive development

For peer review only

Abstract

Introduction: Thiamine (vitamin B1) deficiency remains a concern in Cambodia where women with low thiamine intake produce thiamine-poor milk, putting their breastfed infants at risk of impaired cognitive development and potentially fatal infantile beriberi. Thiamine fortification of salt is a potentially low-cost, passive means of combating thiamine deficiency, however both the dose of thiamine required to optimize milk thiamine concentrations, as well as usual salt intake of lactating women, are unknown.

Methods and analysis: In this community-based randomized controlled trial, 320 lactating women from Kampong Thom, Cambodia will be randomized to one of four groups to consume one capsule daily containing 0, 1.2, 2.4, or 10 mg thiamine as thiamine hydrochloride, between 2 and 24 weeks postnatal. The primary objective is to estimate the dose where additional maternal intake of thiamine no longer meaningfully increases infant thiamine diphosphate concentrations (ThDP) 24 weeks postnatally. At 2, 12, and 24 weeks we will collect sociodemographic, nutrition, and health information, a battery of cognitive assessments, maternal (2 & 24 weeks) and infant (24 weeks only) venous blood samples (biomarkers: ThDP and transketolase activity), and human milk samples (also at 4 weeks; biomarker: milk thiamine concentrations). All participants and their families will consume study-provided salt *ad libitum* throughout the trial, and we will measure salt disappearance each fortnight. Repeat weighed salt intakes and urinary sodium concentrations will be measured among a sub-set of 100 participants. Parameters of E_{max} dose-response curves will be estimated using non-linear least squares models with both 'intention to treat' and a secondary 'per-protocol' (capsule compliance ≥80%) analyses.

Ethics and dissemination: Ethical approval was obtained in Cambodia (National Ethics Committee for Health Research 112/250NECHR), Canada (Mount Saint Vincent University Research Ethics Committee 2017-141), and the United States (University of Oregon Institutional Review Board 07052018.008). Results will be shared with participants' communities, as well as relevant government and scientific stakeholders via presentations, academic manuscripts, and consultations. ClinicalTrials.gov Identifier: NCT03616288.

Strengths and limitations of this study

- Human milk is thiamine-responsive, however this is the first study to assess the dose of thiamine required to optimize human milk thiamine concentrations, vital information required to inform fortification programs (minimize costs and unnecessary overages).
- Previous work has identified delayed cognitive development among children with low thiamine intake in early life, however this will be the first study to explore cognitive development among infants with controlled thiamine exposures in the first 6 months postnatal.
- We will collect mother's blood, human milk, and infant's blood at 24 weeks postnatal, enabling a robust evaluation of associations between maternal supplementation and these biomarkers.
- The study intervention begins at 2 weeks postnatal, so while randomization should prevent pre-treatment differences between treatment groups, we will not have biochemical thiamine status of the mother during pregnancy, nor the infant at birth.

- Household salt disappearance measures may over- or under-estimate actual usual salt intake as it will not account for wastage, nor salt from food consumed outside the home (e.g. with relatives, purchased at market), respectively.

For peer review only

Background

Thiamine (vitamin B_1) is an essential water-soluble micronutrient required for energy metabolism and nerve impulse conduction [1,2]. Thiamine deficiency was recently called the forgotten disease of Asia [3], as it remains an under-studied public health issue despite evidence of suboptimal thiamine intake or status in the region [4], including Cambodia [5–11]. Thiamine deficiency is of particular concern among lactating women because mothers with poor dietary thiamine intake and/or status produce milk low in thiamine, putting their exclusively breastfed infants at a high risk of developing infantile beriberi [12]. Infantile beriberi presents during the exclusive breastfeeding period and without treatment can result in death within hours of clinical presentation [4,13]. In addition, a growing body of evidence from an unfortunate 'natural experiment' in which thiamine was erroneously omitted from infant formula [14], suggests that thiamine deficiency not severe enough to cause clinical beriberi symptoms can negatively impact cognitive development and functioning [15–17]. To date the precise pathways and mechanisms remain poorly understood, and important questions remain about the timing and levels of deficiency, as well as the ability of prophylactic or therapeutic interventions to prevent or remediate the effects of suboptimal thiamine status on cognitive outcomes in humans [18].

Thiamine is found in pork, whole grains, and legumes [1], and must be consumed routinely due to the relatively short half-life and lack of body storage [4]. Lactating women have an estimated average requirement (EAR) and recommended dietary allowance (RDA) for thiamine of 1.2 and 1.4 mg/day, respectively [19]. For infants aged 0-6 months, an adequate intake (AI) of 0.2 mg/day was derived using the human milk thiamine concentrations from healthy, well-nourished mothers, assuming a daily milk intake of 780 mL [19].

For peer review only

The dietary staple of Cambodia is B-vitamin poor, white, polished rice, which accounts for upwards of 60% of daily energy intake [20]. Given the challenges associated with supplementation and changing dietary patterns (recently reviewed in [4]), fortification, a sustainable, cost-effective, and passive intervention [21–24], is a potentially suitable solution for improving maternal thiamine intake [23]. Thiamine has few technical constraints as a fortificant [21], and there is no tolerable upper intake level (UL) for thiamine because there have been no reports of adverse effects of excess thiamine intake [19,25,26]. We recently demonstrated that maternal consumption of thiamine-fortified fish sauce significantly increased maternal, human milk, and infant thiamine status [27]. However, centrally produced fish sauce may not reach the poorest communities who make their own fish sauce, and consumption of this condiment is not universal in all regions where we find thiamine deficiency [28,29]. Conversely, salt is a common condiment in most regions of the world, and has proven to be a successful global fortification vehicle for iodine [30,31].

The overall aim of this study is to obtain the information necessary to formulate a thiamine-fortified salt for future use in Cambodia (ideal thiamine dose, usual salt intake), and to explore the impact of various doses of maternal thiamine on markers of infant cognitive development.

Study outcomes and analysis objectives

Primary Outcome and Analysis Objective: To estimate the dose on the dose response curve where additional maternal intake of thiamine (oral dose) no longer meaningfully increases human milk total thiamine concentration at 24 weeks postpartum.

For peer review only

Secondary Outcomes and Analysis Objectives:

1. To estimate the dose on the dose response curve where additional maternal intake of thiamine (oral dose) no longer meaningfully increases infant thiamine diphosphate concentrations (ThDP) 24 weeks postnatally, and assess whether this depends on the presence/absence of a genetic hemoglobin disorder.
2. To estimate the dose on the dose response curve where additional maternal intake of thiamine (oral dose) no longer meaningfully increases human milk total thiamine concentration at 4 and 12 weeks postpartum.
3. To estimate the dose on the dose response curve where additional maternal intake of thiamine (oral dose) no longer meaningfully increases infant transketolase activity at 24 weeks postnatally, and assess whether this depends on the presence/absence of a genetic hemoglobin disorder.
4. To estimate the dose on the dose response curve where additional maternal intake of thiamine (oral dose) no longer meaningfully increases maternal ThDP at 24 weeks postpartum, and assess whether this depends on the presence/absence of a genetic hemoglobin disorder.
5. To estimate the dose on the dose response curve where additional maternal intake of thiamine (oral dose) no longer meaningfully increases maternal transketolase activity at 24 weeks postpartum, and assess whether this depends on the presence/absence of a genetic hemoglobin disorder.

For peer review only

6. To test for differences between the 4 randomized groups on human milk total thiamine at 4, 12, and 24 weeks postpartum.
7. To test for differences between the 4 randomized groups on maternal ThDP at 24 weeks postpartum, and assess whether this depends on the presence/absence of genetic hemoglobin disorder.
8. To test for differences between the 4 randomized groups on maternal transketolase activity at 24 weeks postpartum and assess whether this depends on the presence/absence of a genetic hemoglobin disorder.
9. To estimate usual household salt intake from mean fortnightly salt disappearance (weight lost, in g).
10. To estimate salt intake among a subset of 100 lactating women, their male partners (if applicable), and their children 24-59 months (if applicable) using observed weighed salt intake records.
11. To estimate sodium intake using 24 hr urinary sodium concentrations among a subset of 100 lactating women.
12. To test for differences between infants in the 0 & 10 mg randomized groups on Composite Mullen and the 5 subscales of the Mullen at 24 weeks postnatally.
13. To test for differences between infants in the 0 & 10 mg randomized groups on Visual Paired Comparison Novelty Score and the attention and processing speed subscales at 24 weeks postnatally.
14. To test for differences between infants in the 0 & 10 mg randomized groups on the Language Preference Task Score at 24 weeks postnatally.

For peer review only

15. To test for differences between infants in the 0 & 10 mg randomized groups on oculomotor scores at 24 weeks postnatally.
16. To determine the effect of inflammation, as measured by C-reactive protein (CRP) and α-1-acid-glycoprotein (AGP) on maternal ThDP at 2 and 24 weeks postpartum, and infant ThDP at 24 weeks postnatal.

Methods

Study design and setting

This is a double-blind, four-parallel arm randomized controlled trial among lactating women and their newborn infants. The study timeline and data collection schedule can be found in **Figure 1**. The study is community-based, with data collection taking place in women's homes in Kampong Thom province in central Cambodia. All women live rurally, in villages in the catchment areas of the following health centres: Tboung Kapoeur, Kampong Svay, Sankor, Chey, Salavisai, Prey Kuy, Prey Pros, and Srayov.

Eligibility criteria

All women must provide written informed consent to participate. Participants must meet the following criteria:

- mothers of a newborn;
- aged 18-45 years;
- most recent pregnancy was normal (i.e. no known chronic conditions, preeclampsia, gestational diabetes etc.), and the singleton infant was born without complications (e.g. low birth weight (<2.5 kg), tongue tie, cleft palate);

For peer review only

- intends to exclusively breastfeed for six months;
- resides in Kampong Thom province, Cambodia, and is not planning to move in the next six months;
- is not currently taking, and hasn't taken any thiamine-containing supplements over the previous 4 months;
- is not currently participating in any nutrition programs beyond normal care;
- is willing to consume one capsule daily from 2 weeks through 24 weeks postpartum;
- is willing for her entire household consume only salt provided by the study team; and
- is willing for the following biological samples to be collected: a maternal venous blood sample and human milk sample at 2 weeks postpartum, a human milk sample at 4 and 12 weeks postpartum, and maternal and infant blood samples and a human milk sample at 24 weeks postpartum.

Recruitment, allocation, randomization, and blinding

A participant flow diagram is shown in **Figure 2**. Participants will be recruited through antenatal care visits, as well as through consultation with local village chiefs, elders, and health centre staff. Pregnant women will be advised and provided with a general overview of the research study, and then the names and contact information of interested women will be shared with the research assistants, who follow up after delivery to screen for eligibility, obtain consent, and enroll women. Recruitment will continue on a rolling basis until 320 women are enrolled.

Women will be randomly assigned to one of the four treatment groups as described in **Table 1**. Participants, research assistants, study investigators, and data analysts will be blinded to the

For peer review only

randomized groups. The randomization will be stratified by health centre and use randomly permuted blocks of size 8 within strata to assign participants to one of eight treatment codes in the ratio 1:1:1:1:1:1:1:1 (2 treatment codes per treatment group to assist with blinding). A computer-generated randomization schedule was prepared by the study statisticians using ralloc.ado in Stata (College Station, TX, USA). An independent scientist determined which treatment code corresponded to which treatment group. When a participant is enrolled in the study, the research assistants open an envelope labeled with the study ID that contains the study ID and accompanying treatment code that will be assigned to that participant.

Table 1: Treatment arms for the *Trial of thiamine supplementation in Cambodia.*

Treatment Arm	Thiamine Dose	Rationale
Negative Control	0 mg/day	Negative Control (placebo)
EAR	1.2 mg/day	1 x thiamine EAR for lactating women [19]
Double EAR	2.4 mg/day	2 x thiamine EAR for lactating women [19]
Positive Control	10 mg/day	Positive Control (dose currently given in supplemental form in Myanmar) [4]

EAR, estimated average requirement

Intervention

Women will be asked to consume one capsule daily between 2 and 24 weeks postnatal. The intervention is opaque capsules containing varying amounts of thiamine hydrochloride and cellulose filler, as indicated in **Table 1**. All thiamine is delivered as thiamine hydrochloride, calculated using a 1.271 correction factor (ratio of molecular weights of thiamine hydrochloride and thiamine). Capsules were formulated, compounded, and packaged as 14-day blister packs at the Quinpool Wellness Centre in Halifax, Nova Scotia, Canada.

For peer review only

All capsules were formulated and packed in two batches in July and November 2018. Thiamine content was assessed by an independent lab (USANA, Salt Lake City, UT, USA) before distribution to participants, and will continue to be assessed bi-monthly for the duration of the study. Capsules will be deemed acceptable if the average thiamine concentration for each code (n=10 samples per code) falls within ±15% of the values shown in **Table 1**.

Compliance

Compliance will be assessed fortnightly: research assistants will visit the participant's home to collect the old blister pack and complete a capsule count, and to deliver a new blister pack. A woman will be considered compliant if she consumes ≥80% capsules over the 22-week intervention.

Participant and public involvement in research

This research is being conducted in collaboration with the Cambodian Ministry of Health's National Maternal and Child Nutrition Programme, the Cambodian Ministry of Planning's Sub-Committee for Food Fortification, and Kampong Thom province's Provincial Health Department and Health Operational District. Participants and the public will be involved in the dissemination of study results (public village meetings to share findings), however were not involved in research question development, nor the design of the study or intervention.

Data and biological sample collection

Data and biological samples will be collected at enrolment (between t=0 to 2 weeks postpartum), at 2, 4, 12, and 24 weeks postnatal, as well as during fortnightly monitoring visits (see **Figure 1**).

For peer review only

Chart review

Upon enrolment, a healthcare worker in the Health Centre will record data from the dyad's chart such as any interventions/events at birth, maternal age, number of antenatal care visits, number of iron-folic acid tablets consumed, time and date of birth, sex of infant, and infant anthropometrics.

Questionnaires

Using an interviewer-administered questionnaire, we will collect demographic and socioeconomic information, health information including questions about sleep (adapted from [32]) and postpartum depression [33], limited dietary intake data (e.g. perceptions of salt intake, postpartum food taboos [34]), and infant and young child feeding (IYCF) knowledge and behaviors at 2, 12, and 24 weeks postnatal.

Anthropometry

Anthropometric measures are as follows: infants: length, weight, and head circumference; mothers: height and weight. Initial measurements will be taken in the Health Centre at delivery, and all other measurements will be collected in participant's homes (2, 12, and 24 weeks postnatal) using calibrated instruments and standard protocols as per [35].

Fortnightly monitoring

Every two weeks research assistants will visit the participant's home to distribute a new blister pack and assess intervention compliance. They will also administer a short questionnaire about

For peer review only

selling or sharing of salt, IYCF practices, and the household members eating from the common household pot.

Salt disappearance

At the initial home visit after randomization, research assistants will check that the household has removed all salt from the home, and will distribute table salt in specialized study containers. Using calibrated scales (1 g graduation), research assistants will log the initial weight of the salt container(s). Participants will be asked to consume only salt provided, and since Cambodian families eat from a common pot, they will be instructed for all family meals to be prepared using this salt. Salt disappearance will continue to be assessed at fortnightly visits.

We will also collect supplemental information in a subset of households (*n*=100) using observed weighed intakes and maternal urinary sodium concentrations (randomly selected from larger study, with a second day repeat on non-consecutive days within 1 week of initial visit). The participant (mother), the husband/man in household aged 18-50 years, and a child between 24-59 months (husband/man and child will not be applicable in all households) will be enrolled in this sub-study. Research assistants will sit in the participants home from dawn through dusk, recording, at the individual level, intake for the 1-3 individuals for all table salt and salt-containing condiments (e.g. fish and soy sauces) consumed. For the mothers only, we will assess 24-hour urinary sodium concentrations [25]. The mother will discard her first urine upon awakening, record the time, and then collect all urine over the next 24 hours into a provided container; the collection will end after the woman’s first void the following morning. The research assistants will visit her home to weigh the full container and then take aliquots for

For peer review only

testing. The urine sample will be temporarily stored at -20°C in Kampong Thom for <2 weeks before being transported to the National Institute for Public Health Laboratory (NIPHL) in Phnom Penh for urinary sodium analysis on a EasyLtye Na/K/Cl Analyser (Medica, Dusseldorf, Germany) and urinary creatinine assessment using a Kenza 240TX Analyser (Biolabo Diagnostics, Maizy, France). We will model thiamine fortification of salt using the University of Iowa's Intake Monitoring, Assessment and Planning Program software (http://www.side.stat.iastate.edu/imapp.php).

Venous blood samples

Trained, Khmer-speaking nurses will meet mothers and infants at their home or a central village location (health centre, or village chief's home) to collect maternal and infant blood samples into EDTA-coated tubes. Maternal blood samples (9 mL) will be collected at 2 and 24 weeks postnatal; infant samples (5 mL) will be collected only at 24 weeks postnatal. Time of day and time since last meal will be recorded.

Human milk samples

Human milk samples will be collected using a battery-powered single breast pump (Swing Breast pump, Medela) at 2, 4, 12, and 24 weeks postnatal. One full breast expression (single breast) will be collected from the breast women self-identify as being more 'full' (the breast not most recently emptied). Since time of day has little effect on milk thiamine concentrations [36], samples can be collected at any time of day, however, time of day, time since last meal, and breast side will be recorded.

For peer review only

Blood and human milk processing

Biological samples will be collected in the village, placed on ice, and transported to the field lab in Kampong Thom within 5 hours of collection for processing. All samples will be stored at -20°C for <2 weeks before being transported the NIPHL for storage at -80°C. Samples will be batch-shipped on dry ice after the 24 weeks postnatal data collection is completed.

Blood and human milk analysis

Venous blood samples will undergo analysis for both transketolase activity and thiamine diphosphate concentrations (ThDP) at the NIHR BRC Nutritional Biomarker Laboratory at the University of Cambridge in the United Kingdom. At 2 weeks postnatal, maternal whole blood ThDP and erythrocyte transketolase activity will be measured. At 24 weeks postnatal, ThDP and transketolase activity will be measured in both whole blood and erythrocytes, and infant samples will be assayed for whole blood ThDP and erythrocyte transketolase activity. When ThDP is assessed in whole blood, concentrations must be normalized to hemoglobin concentrations and/or hematocrit [37] measured via a HemoCue 201 portable hemoglobinometer and capillary hematocrit tubes, respectively.

Evidence from the 2014 Cambodian National Micronutrient survey indicates that eThDP is affected by the presence/absence of genetic hemoglobin disorders (unpublished data). With this, it is vital to assess genetic hemoglobin disorders, as this could influence participant's response to thiamine, it may influence transketolase, and it could change thiamine dosage requirements in other countries. Therefore, at one timepoint only (2 weeks postnatal for maternal, and 24 weeks postnatal for infant samples), samples will undergo hemoglobin capillary electrophoresis

For peer review only

analysis to identify structural hemoglobin variants [38]. Buffy coat samples will be stored in the BioBank in the Department of Applied Human Nutrition at Mount Saint Vincent University in Halifax, Nova Scotia, Canada, for potential later assessment of genetic hemoglobin disorders.

Biomarkers may be influenced by inflammation, which is common in this population [39,40]. Plasma samples will be sent to Dr. Jurgen Erhardt at the VitMin Lab in Germany for analysis of C-reactive protein (CRP) and α-1-acid-glycoprotein (AGP) using an immunosorbent assay [41]. Note that this assay will also measure retinol binding protein (RBP), ferritin, and soluble transferrin receptor (sTfR). Other plasma samples will be stored in the BioBank for potential future use.

The weight of the full human milk expression will be recorded, the sample mixed, and 2 mL aliquots obtained in amber cryovials. Human milk thiamine concentrations will be measured at the USDA/ARS Western Human Nutrition Research Center, University of California, Davis [42]. Additional human milk samples will be stored in the BioBank for potential future use.

Cognitive assessments

We will conduct cognitive assessments at 2, 12, and 24 weeks postnatal; see **Figure 1** for battery of assessments at each timepoint. Assessments include: the Hammersmith Infant Neurological Exam, Infant Mullen Scales of Early Learning, a Primary Engagement Task, a Secondary Engagement Task, the Caregiver Reported Early Development Instruments (CREDI), an oculomotor exam, the Visual Paired Comparison task, and the Language Preference task.

For peer review only

The Hammersmith Infant Neurological Exam is a standard tool for providing basic information about infants' neurological status through use of gentle touch and gentle social interaction in order to examine infants' sensory and motor responses; it has previously shown excellent interobserver reliability even among less experienced staff [43]. The Infant Mullen Scales of Early Learning is an individually administered, multi-domain measure of early development, with scales measuring development in visual reception, fine motor, gross motor, receptive language, and expressive language [44]. The Primary Engagement Task measures individual variation in infant, caregiver, and dyad-level developmental change in the ability to engage contingently with the partner in direct positive mutual engagement interactions. The Secondary Engagement Task measures individual variation in caregiver and infant ability to engage mutually in relation to an external object. The CREDI is a caregiver-reported measure of child development that has been shown to have right validity and reliability in low-resource settings [45]. An oculomotor exam has been included because oculumotor disturbances are not uncommon among thiamine deficient adults presenting with Wernicke's encephalopathy [46]. The Visual Paired Comparison task probes infants' recognition memory and attention, and has been used to predict subsequent verbal IQ in middle childhood [47,48], and produce measures of attention predictive of developmental maturity and better cognitive function [49,50]. The Language Preference Task is designed to measure individual variation in infants' level of interest in the kind of language that caregivers typically direct toward infants (aka infant-directed talk or 'motherese') relative to a) the kind of language that is typically directed toward adults (aka adult-directed talk), and b) sounds that are matched in complexity but are non-linguistic (non-linguistic analog). Over a series of trials, infants hear these three different kinds of sound samples through an audio speaker (infant-directed talk, adult-directed talk, non-linguistic analog), and the

For peer review only

duration that they look toward a visual display during each sound sample is subsequently measured from the videotaped record collected during the session.

Participant remuneration

All participants will receive a mobile phone and mobile phone credits. In addition, modest, study-appropriate remuneration such as a sarong, canned fish, or laundry soap will be provided at biological sample collection points (2, 4, 12, and 24 weeks postnatal, and after urine collection).

Safety considerations, safety monitoring, and breaking the blinding

There is no UL for thiamine [19,25,26], and therefore no necessity for a Serious Adverse Events Committee or a Data Safety Monitoring Board in the current study. While we are confident that there is a very low risk of serious adverse events in this study related to the intervention, we do expect some infant deaths in our cohort due to other causes. Per the 2014 Cambodian Demographic and Health Survey rates of neonatal (birth to one month) and infant (birth to one year) mortality [51], we calculated an expected mortality rate in our study to lie between 1.67 and 3.68 infant deaths.

The master ID list will not be unblinded during the study unless in the unlikely event of an adverse event in the trial. If there is a medical emergency and unblinding of a participant is required, our blinding mechanism would allow for only one of the two codes per treatment arm to be revealed (as each treatment arm will have two codes). We would then document and report to the funding agency and all ethics committees why any premature unblinding occurred.

For peer review only

Once data collection and cleaning is complete, the database will be locked and unblinded treatment codes will be included in the database because a blinded analysis is not possible for estimating dose response curves.

Sample size calculations

To detect a clinically meaningful difference of 40 μg/L in human milk total thiamine concentration between any two treatment groups with 90% power, assuming a SD of 43 μg/L (estimated SD of control group in a Cambodian thiamine-fortified fish sauce trial [52]), 48 women are required per treatment group, or a total of 192 women. This sample size allows for 20% attrition and uses a two-sided alpha of 0.0083 for each of the 6 pairwise comparisons between the four treatment groups in order to control the familywise error rate at the 0.05 level using a Bonferroni adjustment for multiple comparisons. Recruitment of 320 participants (80 per group) was planned to allow for some uncertainty in the assumed values, particularly the SD which may be larger than anticipated.

Simulations of 500 dose-response curves for human milk total thiamine concentration were conducted to estimate the precision that this sample size would provide for addressing the primary study objective (i.e. for estimating the dose on the dose response curve where additional maternal intake of thiamine (oral dose) no longer meaningfully increases human milk total thiamine concentration at 24 weeks postpartum, defined as the dose required to achieve 90% of the average maximum human milk total thiamine concentration). Data were simulated based on an average minimum and maximum concentration of 136 μg/L [52] and 210 μg/L [19], respectively, with a SD of 43 μg/L [52], and reaching 50% and 90% of the maximum average

For peer review only

concentration at dose 1.2 mg/d (EAR group) and 2.4 mg/d (double EAR group) respectively. Assuming an E_{max} dose-response curve, the precision of the estimated dose is ±1.29 mg/d (i.e. the 95% confidence interval for the estimated dose will be within ±1.29 mg/d of the point estimate).

Data and sample management and security

Participants will be given a unique alpha-numeric study ID code, not derived from personal identifiers; this code will link all data collected from this individual. All data will be collected directly on tablets using structured forms designed specifically for this study. Data will be reviewed daily by the research assistant and field supervisor before secure download and integration with the larger database. Data management and security procedures, including assurance of confidentiality, adhere to the Canadian Tri-Council Policy Statement on Ethical Conduct for Research Involving Humans (TCPS2 CORE) guidelines, and are outlined in full in the protocol at clinicaltrials.gov (NCT03616288).

Data analysis plan

A full statistical analysis plan is published in the clinicaltrials.gov entry (NCT03616288). The primary analyses will be performed on an ‘intention to treat’ basis, where all participants will be analyzed according to their allocated treatment group regardless of compliance. A secondary ‘per-protocol’ analysis will be performed on the subset of women who consumed ≥80% capsules over the study period. The parameters of the E_{max} dose-response curves will be estimated using non-linear least squares models. Separate curves will be fitted for outcomes measured at 4 weeks, 12 weeks, and 24 weeks postnatal. The dose where additional maternal intake of thiamine

For peer review only

no longer meaningfully increases milk thiamine concentration, defined as the dose that achieves 90% of the maximum concentration, will be estimated from the fitted curve with standard errors estimated by bootstrapping for calculating 95% confidence intervals.

Comparisons between treatment groups will be performed using linear mixed-effects models to account for repeated measurements and adjusted for randomization strata and levels at 2 weeks postnatal. Interaction tests will be performed to assess whether treatment effects depend on genetic hemoglobin disorder. Missing data will be addressed using multiple imputation to create 100 complete datasets for analysis, with a sensitivity analysis performed on the raw (unimputed) data [53].

Ethics and dissemination

Approvals were obtained from the following Research Ethics Boards: the National Ethics Committee for Health Research, Cambodia (112/250NECHR), Mount Saint Vincent University Research Ethics Committee, Canada (2017-141); and the University of Oregon Institutional Review Board, USA (07052018.008). This study is registered at clinicaltrials.gov (ClinicalTrials.gov Identifier NCT03616288 [August 7, 2018]; Protocol version 2.1 [July 24, 2018]).

Updates on the study will be shared at the monthly Cambodian Nutrition Working Group meetings, attended by researchers and non-governmental organizations (NGO) engaging in

For peer review only

nutrition research and programming in Cambodia. Scaling Up Nutrition and other relevant groups will also be updated regularly. Research results will be presented at academic nutrition, public health, and psychology conferences, and in peer-reviewed journals that offer open access. Data will be made available on a public repository after dissemination.

We plan to create various lay outputs from this study that can be used by NGO and government agencies working with families, or can be accessed directly by families. We will host a Dissemination Workshop in Phnom Penh open to relevant stakeholders (NGOs, government, researchers, media, clinicians, public health, all sectors) to share the main outcomes of the study, and officially 'launch' lay resources. We will also return to each community in Kampong Thom to conduct a village-wide meeting relaying study results.

Trial status

Participant recruitment began in August 2018, and data collection started September 12, 2018. We estimate data collection will be completed by June 2019.

Discussion

Suboptimal maternal thiamine intake and/or status puts exclusively breastfed infants at risk of low thiamine status [12], impaired cognitive development [15–17], and infantile beriberi, which can be fatal [13]. Thiamine fortification of salt is a potentially low-cost and sustainable means of combating suboptimal thiamine status, however knowledge gaps must be filled before thiamine

For peer review only

fortification can proceed [4]. There are limited data available on the dose of thiamine required by lactating women to optimize the thiamine concentrations in their milk. Further, usual salt intake among lactating women is unknown. Finally, although there is emerging evidence that low thiamine intake in early life impacts cognitive development, this has yet to be assessed in a controlled research environment. This study is posed to address these knowledge gaps and provide valuable information to inform any potential future thiamine fortification efforts in Cambodia or bordering countries.

Acknowledgements

We thank the members of the study's Scientific Advisory Board for valuable input on the study protocol (alphabetical order): Drs. Megan Bourassa, Levente Diosady, Lisa Houghton, Arnaud Laillou, and Annie Wesley. We thank Dr. Ken Brown for assistance and insight during study conception, Dr. Geraldine Richmond for her role in early protocol development, and Keith Porter for early input on project feasibility and implementation. We acknowledge USANA (Salt Lake City, UT, USA) for the generous provision of laboratory analysis for study capsule thiamine hydrochloride content.

Author's Contributions

KCW drafted the manuscript. KCW, HK, TJG, FW, JRM, and DB conceived the study and wrote the initial study protocol. MB, SP, LY, SL, KC, & JG assisted in developing the protocol. HK, MB, & PS facilitated implementation of the study. LY & SL developed the statistical analysis plan. KC & JG are involved in study coordination. All authors participated in, read, and approved the final manuscript.

For peer review only

Funding

This study is funded through the Bill & Melinda Gates Foundation and the Sackler Institute for Nutrition Science, New York Academy of Sciences (Opportunity ID OPP1176128) as 'Objective 3: Trial of thiamine supplementation in Cambodia', as part of the larger program grant entitled 'Improving estimates of the global burden of thiamine deficiency disorders (TDDs) and approaches to their control'. The study funders were involved in early study conception; funders will have no involvement in the study design, collection, management, analysis, and interpretation of data, writing of the report, nor the decision to submit the report for publication. LNY was supported by an Australian National Health and Medical Research Council Early Career Fellowship (ID 1052388).

Completing Interest Statement

None to declare.

List of Figures

Figure 1: Study timeline and data collection schedule for *Trial of thiamine supplementation in Cambodia.*

Figure 1 legend. CREDI: Caregiver Reported Early Development Instruments; wk: week.

Figure 2: Participant flow chart for *Trial of thiamine supplementation in Cambodia.*

Figure 2 legend. Double EAR: Double EAR group (2.4 mg thiamine as thiamine hydrochloride); EAR: EAR group (1.2 mg thiamine as thiamine hydrochloride); Placebo:

For peer review only

negative control group (placebo; 0 mg thiamine); Positive Control: Positive Control group (10 mg thiamine as thiamine hydrochloride).

For peer review only

References

1 Bemeur C, Butterworth RF. Thiamin. In: Ross AC, Calallero B, Cousins RJ, *et al.*, eds. *Modern Nutrition in Health and Disease*. Baltimore, MD: Lippincott Williams & Wilkins 2014. 317–24.

2 Fattal-Valevski A. Thiamine (Vitamin B1). *J Evid Based Complementary Altern Med* 2011;**16**:12–20. doi:10.1177/1533210110392941

3 Khounnorath S, Chamberlain K, Taylor AM, *et al.* Clinically unapparent infantile thiamin deficiency in Vientiane, Laos. *PLoS Negl Trop Dis* 2011;**5**:e969. doi:10.1371/journal.pntd.0000969

4 Whitfield KC, Bourassa MW, Adamolekun B, *et al.* Thiamine deficiency disorders: diagnosis , prevalence , and a roadmap for global control programs. *Ann N Y Acad Sci* 2018;**1430**:3–43. doi:10.1111/nyas.13919

5 Whitfield KC, Karakochuk CD, Liu Y, *et al.* Poor thiamin and riboflavin status is common among women of childbearing age in rural and urban Cambodia. *J Nutr* 2015;**145**. doi:10.3945/jn.114.203604

6 Whitfield KC, Green TJ. Erratum for Whitfield et al. Poor thiamin status is common among women of childbearing age in rural and urban Cambodia. J Nutr 2015;145:628-33. *J Nutr* 2016;**146**:147–8. doi:10.3945/jn.115.225599.147

7 Coats D, Shelton-Dodge K, Ou K, *et al.* Thiamine deficiency in Cambodian infants with and without beriberi. *J Pediatr* 2012;**161**:843–7.

8 Whitfield KC, Karakochuk CD, Kroeun H, *et al.* Perinatal consumption of thiamine-fortified fish sauce in rural Cambodia: A randomized clinical trial. *JAMA Pediatr* 2016;**170**. doi:10.1001/jamapediatrics.2016.2065

9 Keating EM, Nget P, Kea S, *et al.* Thiamine deficiency in tachypnoeic Cambodian infants. *Paediatr Int Child Health* 2014;**35**:312–8. doi:10.1179/2046905514Y.0000000162

10 Porter SG, Coats D, Fischer PR, *et al.* Thiamine deficiency and cardiac dysfunction in Cambodian infants. *J Pediatr* 2014;**164**:1456–61. doi:10.1016/j.jpeds.2014.01.049

11 Whitfield KC, Smith G, Chamnan C, *et al.* High prevalence of thiamine (vitamin B1) deficiency in early childhood among a nationally representative sample of Cambodian women of childbearing age and their children. *PLoS Negl Trop Dis* 2017;**11**. doi:10.1371/journal.pntd.0005814

12 Allen LH. B vitamins in breast milk: relative importance of maternal status and intake, and effects on infant status and function. *Adv Nutr* 2012;**3**:362–9.

13 Luxemburger C, White NJ, ter Kuile F, *et al.* Beri-beri: the major cause of infant mortality in Karen refugees. *Trans R Soc Trop Med Hyg* 2003;**97**:251–5.

14 Fattal-Valevski A, Kesler A, Sela B-A, *et al.* Outbreak of life-threatening thiamine deficiency in infants in Israel caused by a defective soy-based formula. *Pediatrics* 2005;**115**:233–8. doi:10.1542/peds.2004-1255

15 Harel Y, Zuk L, Guindy M, *et al.* The effect of subclinical infantile thiamine deficiency on motor function in preschool children. *Matern Child Nutr* 2017;e12397. doi:10.1111/mcn.12397

16 Fattal-Valevski A, Azouri-Fattal I, Greenstein YJ, *et al.* Delayed language development due to infantile thiamine deficiency. *Dev Med Child Neurol* 2009;**51**:629–34. doi:10.1111/j.1469-8749.2008.03161.x

17 Fattal I, Friedmann N, Fattal-Valevski A. The crucial role of thiamine in the development of syntax and lexical retrieval: a study of infantile thiamine deficiency. *Brain*

For peer review only

2011;**134**:1720–39. doi:10.1093/brain/awr068

18 Prado EL, Dewey KG. Nutrition and brain development in early life. *Nutr Rev* 2014;**72**:267–84. doi:10.1111/nure.12102

19 Institute of Medicine. Thiamin. In: *Dietary Reference Intakes for thiamin, riboflavin, niacin, vitamin B6, folate, vitamin B12, pantothenoc acid, biotin, and choline*. Washington, DC: : National Academy Press 1998. 58–86.

20 FAO Statistics Division. Food Balance Sheets: Cambodia (2011). 2014. http://faostat.fao.org/site/368/DesktopDefault.aspx?PageID=368#ancor

21 Allen LH, de Benoist B, Dary O, *et al. Guidelines on food fortification with micronutrients*. Geneva, Switzerland: : World Health Organization and Food and Agriculture Organization of the United Nations 2006.

22 Dwyer JT, Wiemer KL, Dary O, *et al.* Fortification and health: challenges and opportunities. *Adv Nutr* 2015;**6**:124–31. doi:10.3945/an.114.007443.micronutrient

23 Smith G. Micronutrient fortification of food: issues for Asia. *J Nutr Sci Vitaminol (Tokyo)* 2015;**61**:S183-5.

24 Hurrell R. *The Mineral Fortification of Foods*. Surrey, UK: Leatherhead Publishing 1999.

25 Gibson RS. *Principles of Nutritional Assessment*. 2nd ed. New York, NY, USA: Oxford University Press 2005.

26 Smithline HA, Donnino M, Greenblatt DJ. Pharmacokinetics of high-dose oral thiamine hydrochloride in healthy subjects. *BMC Clin Pharmacol* 2012;**12**:4. doi:10.1186/1472-6904-12-4

27 Whitfield KC, Karakochuk CD, Kroeun H, *et al.* Perinatal consumption of thiamine-fortified fish sauce in rural Cambodia: a randomized clinical efficacy trial. *JAMA Pediatr* 2016;**170**:e162065. doi:10.1001/jamapediatrics.2016.2065

28 Nilles EJ, Manaia A, Ruaia B, *et al.* Re-emergence of thiamine deficiency disease in the Pacific islands (2014 – 15): a case-control study. *PLoS One* 2018;**13**:e0198590.

29 Adamolekun B, Hiffler L. A diagnosis and treatment gap for thiamine deficiency disorders in sub-Saharan Africa? *Ann N Y Acad Sci* 2017;**1408**:15–9. doi:10.1111/nyas.13509

30 United Nations Children's Fund (UNICEF). *Improving child nutrition: the achievable imperative for global progress*. New York, USA: 2013. doi:978-92-806-4686-3

31 Andersson M, Takkouche B, Egli I, *et al.* Current global iodine status and progress over the last decade towards the elimination of iodine deficiency. *Bull World Health Organ* 2005;**83**:518–25.

32 Keller MA, Goldbery WA. Co-sleeping: help or hinderance for young children's independence? *Infant Child Dev* 2004;**13**:369–88. doi:10.1002/icd.

33 Gjerdingen D, Crow S, McGovern P, *et al.* Postpartum depression screening at well-child visits: validity of a 2-question screen and the PHQ-9. *Ann Fam Med* 2009;**7**:63–70. doi:10.1370/afm.933.Department

34 Wallace LJ, Summerlee AJS, Dewey CE, *et al.* Women's nutrient intakes and food-related knowledge in rural Kandal province, Cambodia. *Asia Pac J Clin Nutr* 2014;**23**:263–71. doi:10.6133/apjcn.2014.23.2.02

35 Cogill B. Anthropometric Indicators Measurement Guide. Washington, DC: 2003.

36 Hampel D, Shahab-Ferdows S, Islam MM, *et al.* Vitamin concentrations in human milk vary with time within feed, circadian rhythym, and single-dose supplementation. *J Nutr* 2017;**147**:603–11. doi:10.3945/jn.116.242941.1

37 Floridi A, Pupita A, Palmerini C, *et al.* Thiamine pyrophosphate determination in whole

For peer review only

blood and erythrocytes by high performance liquid chromatography. *J Int Vitaminol Nutr* 1983;**54**:165–171.

38 Hartwell SK, Srisawang B, Kongtawelert P, *et al.* Review on screening and analysis techniques for hemoglobin variants and thalassemia. *Talanta* 2005;**65**:1149–61. doi:10.1016/j.talanta.2004.09.013

39 Makurat J, Friedrich H, Kuong K, *et al.* Nutritional and micronutrient status of female workers in a garment factory in Cambodia. *Nutrients* 2016;**8**. doi:10.3390/nu8110694

40 Yong T-S, Chai J-Y, Sohn W-M, *et al.* Prevalence of intestinal helminths among inhabitants of Cambodia (2006-2011). *Korean J Parasitol* 2014;**52**:661–6.

41 Erhardt JG, Estes JE, Pfeiffer CM, *et al.* Combined measurement of ferritin, soluble transferrin receptor, retinol binding protein, and C-reactive protein by an inexpensive, sensitive, and simple sandwich enzyme-linked immunosorbent assay technique. *J Nutr* 2004;**134**:3127–32. doi:134/11/3127 [pii]

42 Hampel D, York ER, Allen LH. Ultra-performance liquid chromatography tandem mass-spectrometry (UPLC-MS/MS) for the rapid, simultaneous analysis of thiamin, riboflavin, flavin adenine dinucleotide, nicotinamide and pyridoxal in human milk. *J Chromatogr B* 2012;**903**:7–13. doi:10.1016/j.jchromb.2012.06.024

43 Maitre NL, Chorna O, Romeo DM, *et al.* Implementation of the Hammersmith Infant Neurological Examination in a high-risk infant follow-up program. *Pediatr Neurol* 2016;**65**:31–8. doi:10.1016/j.pediatrneurol.2016.09.010

44 Mullen EM. Mullen scales of early learning. 1995.

45 McCoy DC, Sudfeld CR, Bellinger DC, *et al.* Development and validation of an early childhood development scale for use in low-resourced settings. *Popul Health Metr* 2017;**15**.

46 Sechi G, Serra A. Wernicke's encephalopathy: new clinical settings and recent advances in diagnosis and management. *Lancet Neurol* 2007;**6**:442–55. doi:10.1016/S1474-4422(07)70104-7

47 McCall RB. What process mediates predictions of childhood IQ from infant habituation and recognition memory? Speculations on the roles of inhibition and rate of information processing. *Intelligence* 1994;**18**:107–25. doi:10.1016/0160-2896(94)90022-1

48 Sigman M, Cohen SE, Beckwith L. Why does infant attention predict adolescent intelligence? *Infant Behav Dev* 1997;**20**:133–40. doi:10.1016/S0163-6383(97)90016-3

49 Rose SA, Feldman JF, Jankowski JJ. Dimensions of cognition in infancy. *Intelligence* 2004;**32**:245–62. doi:10.1016/j.intell.2004.01.004

50 Rose SA, Feldman JF, Jankowski JJ, *et al.* A cognitive cascade in infancy: Pathways from prematurity to later mental development. *Intelligence* 2008;**36**:367–78. doi:10.1016/j.intell.2007.07.003

51 National Institute of Statistics, Directorate General for Health, ICF International. Cambodia Demographic and Health Survey 2014. Phnom Penh, Cambodia and Rockville, Maryland, USA: 2015.

52 Whitfield KC, Karakochuk CD, Kroeun H, *et al.* Household Consumption of Thiamin-Fortified Fish Sauce Increases Erythrocyte Thiamin Concentrations among Rural Cambodian Women and Their Children Younger Than 5 Years of Age: A Randomized Controlled Efficacy Trial. *J Pediatr* 2017;**181**. doi:10.1016/j.jpeds.2016.10.066

53 Graham JW, Olchowski AE, Gilreath TD. How many imputations are really needed? Some practical clarifications of multiple imputation theory. *Prev Sci* 2007;**8**:206–13.

For peer review only

doi:10.1007/s11121-007-0070-9

For peer review only

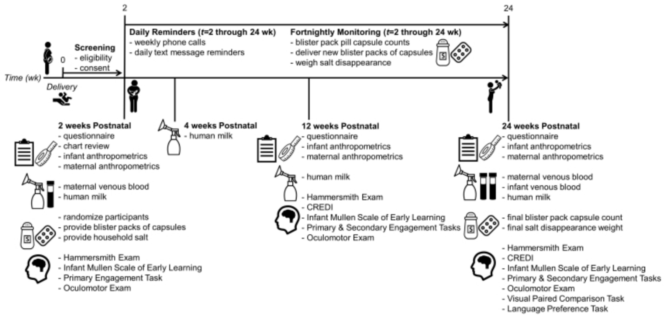

[Caption]: Figure 1: Study timeline and data collection schedule for Trial of thiamine supplementation in Cambodia. [Legend]: CREDI: Caregiver Reported Early Development Instruments; wk: week.

500x190mm (300 x 300 DPI)

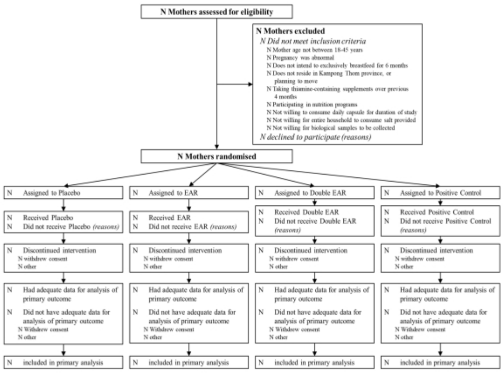

[Caption]: Figure 2: Participant flow chart for Trial of thiamine supplementation in Cambodia.[Legend]: Double EAR: Double EAR group (2.4 mg thiamine as thiamine hydrochloride); EAR: EAR group (1.2 mg thiamine as thiamine hydrochloride); Placebo: negative control group (placebo; 0 mg thiamine); Positive Control: Positive Control group (10 mg thiamine as thiamine hydrochloride).

500x190mm (300 x 300 DPI)

SPIRIT
STANDARD PROTOCOL ITEMS: RECOMMENDATIONS FOR INTERVENTIONAL TRIALS

SPIRIT 2013 Checklist: Recommended items to address in a clinical trial protocol and related documents*

Section/item	Item No	Description	
**Administrative information**			
Title	1	Descriptive title identifying the study design, population, interventions, and, if applicable, trial acronym	P1
Trial registration	2a	Trial identifier and registry name. If not yet registered, name of intended registry	P2,19
	2b	All items from the World Health Organization Trial Registration Data Set	included
Protocol version	3	Date and version identifier	P20
Funding	4	Sources and types of financial, material, and other support	P22
Roles and responsibilities	5a	Names, affiliations, and roles of protocol contributors	P1,22
	5b	Name and contact information for the trial sponsor	P22
	5c	Role of study sponsor and funders, if any, in study design; collection, management, analysis, and interpretation of data; writing of the report; and the decision to submit the report for publication, including whether they will have ultimate authority over any of these activities	P22
	5d	Composition, roles, and responsibilities of the coordinating centre, steering committee, endpoint adjudication committee, data management team, and other individuals or groups overseeing the trial, if applicable (see Item 21a for data monitoring committee)	P16,17, 22
**Introduction**			
Background and rationale	6a	Description of research question and justification for undertaking the trial, including summary of relevant studies (published and unpublished) examining benefits and harms for each intervention	P3-7
	6b	Explanation for choice of comparators	P9
Objectives	7	Specific objectives or hypotheses	P4-7
Trial design	8	Description of trial design including type of trial (eg, parallel group, crossover, factorial, single group), allocation ratio, and framework (eg, superiority, equivalence, noninferiority, exploratory)	P7

For peer review only

For peer review only - http://bmjopen.bmj.com/site/about/guidelines.xhtml

Methods: Participants, interventions, and outcomes

Study setting	9	Description of study settings (eg, community clinic, academic hospital) and list of countries where data will be collected. Reference to where list of study sites can be obtained	P7
Eligibility criteria	10	Inclusion and exclusion criteria for participants. If applicable, eligibility criteria for study centres and individuals who will perform the interventions (eg, surgeons, psychotherapists)	P7-8
Interventions	11a	Interventions for each group with sufficient detail to allow replication, including how and when they will be administered	P9
	11b	Criteria for discontinuing or modifying allocated interventions for a given trial participant (eg, drug dose change in response to harms, participant request, or improving/worsening disease)	P16-17
	11c	Strategies to improve adherence to intervention protocols, and any procedures for monitoring adherence (eg, drug tablet return, laboratory tests)	P10
	11d	Relevant concomitant care and interventions that are permitted or prohibited during the trial	NA
Outcomes	12	Primary, secondary, and other outcomes, including the specific measurement variable (eg, systolic blood pressure), analysis metric (eg, change from baseline, final value, time to event), method of aggregation (eg, median, proportion), and time point for each outcome. Explanation of the clinical relevance of chosen efficacy and harm outcomes is strongly recommended	P6-7
Participant timeline	13	Time schedule of enrolment, interventions (including any run-ins and washouts), assessments, and visits for participants. A schematic diagram is highly recommended (see Figure)	P8-16, Fig2
Sample size	14	Estimated number of participants needed to achieve study objectives and how it was determined, including clinical and statistical assumptions supporting any sample size calculations	P17-18
Recruitment	15	Strategies for achieving adequate participant enrolment to reach target sample size	P8

Methods: Assignment of interventions (for controlled trials)

Allocation:

Sequence generation	16a	Method of generating the allocation sequence (eg, computer-generated random numbers), and list of any factors for stratification. To reduce predictability of a random sequence, details of any planned restriction (eg, blocking) should be provided in a separate document that is unavailable to those who enrol participants or assign interventions	P8-9

For peer review only

Allocation concealment mechanism	16b	Mechanism of implementing the allocation sequence (eg, central telephone; sequentially numbered, opaque, sealed envelopes), describing any steps to conceal the sequence until interventions are assigned	P9
Implementation	16c	Who will generate the allocation sequence, who will enrol participants, and who will assign participants to interventions	P8-9
Blinding (masking)	17a	Who will be blinded after assignment to interventions (eg, trial participants, care providers, outcome assessors, data analysts), and how	P8,17
	17b	If blinded, circumstances under which unblinding is permissible, and procedure for revealing a participant's allocated intervention during the trial	P17
**Methods: Data collection, management, and analysis**			
Data collection methods	18a	Plans for assessment and collection of outcome, baseline, and other trial data, including any related processes to promote data quality (eg, duplicate measurements, training of assessors) and a description of study instruments (eg, questionnaires, laboratory tests) along with their reliability and validity, if known. Reference to where data collection forms can be found, if not in the protocol	P10-16
	18b	Plans to promote participant retention and complete follow-up, including list of any outcome data to be collected for participants who discontinue or deviate from intervention protocols	P11
Data management	19	Plans for data entry, coding, security, and storage, including any related processes to promote data quality (eg, double data entry; range checks for data values). Reference to where details of data management procedures can be found, if not in the protocol	P18-19
Statistical methods	20a	Statistical methods for analysing primary and secondary outcomes. Reference to where other details of the statistical analysis plan can be found, if not in the protocol	P19
	20b	Methods for any additional analyses (eg, subgroup and adjusted analyses)	P19
	20c	Definition of analysis population relating to protocol non-adherence (eg, as randomised analysis), and any statistical methods to handle missing data (eg, multiple imputation)	P19
**Methods: Monitoring**			
Data monitoring	21a	Composition of data monitoring committee (DMC); summary of its role and reporting structure; statement of whether it is independent from the sponsor and competing interests; and reference to where further details about its charter can be found, if not in the protocol. Alternatively, an explanation of why a DMC is not needed	P16-17

For peer review only

	21b	Description of any interim analyses and stopping guidelines, including who will have access to these interim results and make the final decision to terminate the trial	NA
Harms	22	Plans for collecting, assessing, reporting, and managing solicited and spontaneously reported adverse events and other unintended effects of trial interventions or trial conduct	P17
Auditing	23	Frequency and procedures for auditing trial conduct, if any, and whether the process will be independent from investigators and the sponsor	NA
**Ethics and dissemination**			
Research ethics approval	24	Plans for seeking research ethics committee/institutional review board (REC/IRB) approval	P2,20
Protocol amendments	25	Plans for communicating important protocol modifications (eg, changes to eligibility criteria, outcomes, analyses) to relevant parties (eg, investigators, REC/IRBs, trial participants, trial registries, journals, regulators)	P17
Consent or assent	26a	Who will obtain informed consent or assent from potential trial participants or authorised surrogates, and how (see Item 32)	P8
	26b	Additional consent provisions for collection and use of participant data and biological specimens in ancillary studies, if applicable	P8, 14-15
Confidentiality	27	How personal information about potential and enrolled participants will be collected, shared, and maintained in order to protect confidentiality before, during, and after the trial	P18
Declaration of interests	28	Financial and other competing interests for principal investigators for the overall trial and each study site	P22
Access to data	29	Statement of who will have access to the final trial dataset, and disclosure of contractual agreements that limit such access for investigators	P20
Ancillary and post-trial care	30	Provisions, if any, for ancillary and post-trial care, and for compensation to those who suffer harm from trial participation	NA
Dissemination policy	31a	Plans for investigators and sponsor to communicate trial results to participants, healthcare professionals, the public, and other relevant groups (eg, via publication, reporting in results databases, or other data sharing arrangements), including any publication restrictions	P2,20
	31b	Authorship eligibility guidelines and any intended use of professional writers	NA
	31c	Plans, if any, for granting public access to the full protocol, participant-level dataset, and statistical code	P20

For peer review only - http://bmjopen.bmj.com/site/about/guidelines.xhtml

Appendices

Informed consent materials	32	Model consent form and other related documentation given to participants and authorised surrogates	NA
Biological specimens	33	Plans for collection, laboratory evaluation, and storage of biological specimens for genetic or molecular analysis in the current trial and for future use in ancillary studies, if applicable	P12-15

*It is strongly recommended that this checklist be read in conjunction with the SPIRIT 2013 Explanation & Elaboration for important clarification on the items. Amendments to the protocol should be tracked and dated. The SPIRIT checklist is copyrighted by the SPIRIT Group under the Creative Commons "Attribution-NonCommercial-NoDerivs 3.0 Unported" license.

For peer review only

For peer review only - http://bmjopen.bmj.com/site/about/guidelines.xhtml
